# *Pseudomonas aeruginosa* Detection Using Conventional PCR and Quantitative Real-Time PCR Based on Species-Specific Novel Gene Targets Identified by Pangenome Analysis

**DOI:** 10.3389/fmicb.2022.820431

**Published:** 2022-05-04

**Authors:** Chufang Wang, Qinghua Ye, Aiming Jiang, Jumei Zhang, Yuting Shang, Fan Li, Baoqing Zhou, Xinran Xiang, Qihui Gu, Rui Pang, Yu Ding, Shi Wu, Moutong Chen, Qingping Wu, Juan Wang

**Affiliations:** ^1^College of Food Science, South China Agricultural University, Guangzhou, China; ^2^Guangdong Provincial Key Laboratory of Microbial Safety and Health, State Key Laboratory of Applied Microbiology Southern China, Institute of Microbiology, Guangdong Academy of Sciences, Guangzhou, China

**Keywords:** novel target gene, *Pseudomonas aeruginosa*, pangenome analysis, PCR, ready-to-eat vegetables

## Abstract

Mining novel specific molecular targets and establishing efficient identification methods are significant for detecting *Pseudomonas aeruginosa*, which can enable *P. aeruginosa* tracing in food and water. Pangenome analysis was used to analyze the whole genomic sequences of 2017 strains (including 1,000 *P. aeruginosa* strains and 1,017 other common foodborne pathogen strains) downloaded from gene databases to obtain novel species-specific genes, yielding a total of 11 such genes. Four novel target genes, *UCBPP-PA14_00095*, *UCBPP-PA14_03237*, *UCBPP-PA14_04976*, and *UCBPP-PA14_03627*, were selected for use, which had 100% coverage in the target strain and were not present in nontarget bacteria. PCR primers (PA1, PA2, PA3, and PA4) and qPCR primers (PA12, PA13, PA14, and PA15) were designed based on these target genes to establish detection methods. For the PCR primer set, the minimum detection limit for DNA was 65.4 fg/μl, which was observed for primer set PA2 of the *UCBPP-PA14_03237* gene. The detection limit in pure culture without pre-enrichment was 10^5^ colony-forming units (CFU)/ml for primer set PA1, 10^3^ CFU/ml for primer set PA2, and 10^4^ CFU/ml for primer set PA3 and primer set PA4. Then, qPCR standard curves were established based on the novel species-specific targets. The standard curves showed perfect linear correlations, with *R*^2^ values of 0.9901 for primer set PA12, 0.9915 for primer set PA13, 0.9924 for primer set PA14, and 0.9935 for primer set PA15. The minimum detection limit of the real-time PCR (qPCR) assay was 10^2^ CFU/ml for pure cultures of *P. aeruginosa.* Compared with the endpoint PCR and traditional culture methods, the qPCR assay was more sensitive by one or two orders of magnitude. The feasibility of these methods was satisfactory in terms of sensitivity, specificity, and efficiency after evaluating 29 ready-to-eat vegetable samples and was almost consistent with that of the national standard detection method. The developed assays can be applied for rapid screening and detection of pathogenic *P. aeruginosa*, providing accurate results to inform effective monitoring measures in order to improve microbiological safety.

## Introduction

*Pseudomonas aeruginosa* is a common cause of severe nosocomial infections. Patients with metabolic or hematological diseases or patients with malignant immunodeficiency or tumors are especially susceptible to *P. aeruginosa* infection, as are patients in intensive care units (Namaki et al., 2022). *Pseudomonas aeruginosa* is also the most common cause of ventilator-associated pneumonia and burn wound infections, both of which have a mortality rate of >30% (Kidd et al., 2015). Respiratory tract infection with *P. aeruginosa* is a major determinant of the severity of lung disease and is associated with significant incidence rate and mortality of cystic fibrosis (*CF*; Crull et al., 2018; Mesinele et al., 2022).

*Pseudomonas aeruginosa* is widely distributed in water, plants, soil, and humid natural environments, and easily contaminates different kinds of food (Oliver et al., 2015). In addition to being frequently found in bottled mineral water and tap water, *P. aeruginosa* has also been tested positive in ready-to-eat vegetables (Naze et al., 2010; Pelegrin et al., 2021; Ruiz-Roldán et al., 2021). Studies found the ready-to-eat vegetables that were a potential-although rare-vector for colistin- and carbapenem-resistant *P. aeruginosa*, the contamination rate of *P. aeruginosa* has reached 17.5% or 34% (Cai et al., 2015; Hölzel et al., 2018; Kapeleka et al., 2020; Junaid et al., 2021). That is to say, *P. aeruginosa* is a major contaminant of fresh vegetables, which might be a source of infection for susceptible persons within the community (Rahman et al., 2022). Transmission of *P. aeruginosa* along the food chain could cause gastrointestinal infections (Fakhkhari et al., 2022). More importantly, *P. aeruginosa* is the dominant spoilage bacteria and has the strongest spoilage potential in vegetable that are stored under aerobic conditions (Dharmarha et al., 2019; Jin et al., 2021). Additionally, the shelf life of ready-to-eat vegetables is seriously affected by *P. aeruginosa*, which will cause great economic losses (Godova et al., 2020). All told, the presence of *P. aeruginosa* in ready-to-eat vegetables causes food spoilage, reduced shelf life, and economic loss. Therefore, it is necessary to trace the occurrence of potential pollution of this pathogen, so as to provide a scientific basis for ensuring the safety of ready-to-eat vegetables.

Currently, the standard gold method for detecting *P. aeruginosa* in food is the conventional culture method, which is labor-intensive, expensive, and time-consuming (Zhou et al., 2020; Chon et al., 2021). Especially when the number of samples is large, it takes a long time to isolate and identify *P. aeruginosa* from ready-to-eat vegetables by traditional methods (Gharieb et al., 2022). In addition, the traditional culture method determines *P. aeruginosa* according to the green pigment produced by the strain. This method will lead to wrong judgment in actual inspection: one case is that some strains of *P. aeruginosa* do not produce this pigment, which leads to missed inspection. Another situation is that *P. fluorescens* produces the same pigment as *P. aeruginosa*, which makes it impossible to distinguish and cause false positive (Schroth et al., 2018; Junaid et al., 2021). For a long time, scientists have been committed to establishing a rapid and sensitive method for the detection of *P. aeruginosa*, but each method has its advantages and disadvantages (Tang et al., 2017). DNA fingerprinting and 16S DNA-based analyses were used to identify the harm of plant derived *P. aeruginosa* to humans and animals, which is complex and requires very professional inspectors (Ambreetha et al., 2021). Biosensor method and 16r RNA gene amplicon sequencing, which had high detection efficiency, were used to analyze *P. aeruginosa* of food microorganisms, but these methods need complex pretreatment (Zhong et al., 2020; Wind et al., 2021). Illumina whole gene sequencing has great advantages in accuracy, was used to analyze the distribution of *P. aeruginosa* after pasteurized milk, but it takes a lot of testing costs (Maske et al., 2021). Furthermore, 25 articles mentioned health risks from consuming fresh produce by antimicrobial-resistant bacteria, but none quantified the risk (Rahman et al., 2022). When the concentration of *P. aeruginosa* reaches a certain value, it may have the risk of colonization, so it is necessary to quantify its concentration (Kwok et al., 2021). Therefore, it is necessary to develop rapid, accurate, simple, and efficient diagnostic techniques or tools for the detection of *P. aeruginosa* in food, so as to monitor the pollution status and provide scientific basis for the prevention and control of foodborne *P. aeruginosa*.

PCR has been widely employed as a rapid and specific method for the detection of *P. aeruginosa* in a variety of foods and processing environments because of its high specificity, sensitivity, time savings, and easy operation. The target genes *oprL* and *oprI* have been used for the molecular detection of *P. aeruginosa* in burn patients. This approach is a valuable technique for the early and precise detection of *P. aeruginosa* (Jami Al-Ahmadi and Zahmatkesh Roodsari, 2016; Mapipa et al., 2021). A sensitive method has been developed to detect *Pseudomonas pseudomallei* from the soil with PCR by targeting specific flagellin genes (Tungpradabkul et al., 2005). However, most of the reported PCR-based methods for identifying and characterizing *P. aeruginosa* target bacterial virulence genes or 16S and 23S rRNA genes, which provide a limited number of targets (Wei et al., 2015; Wang et al., 2016). With the maturity of whole-genome sequencing technology and the increasing gene pool of new strains, some of the original targets cannot cover the detection of new themes. Therefore, it is vital to mine novel target genes with high species specificity for more accurate and efficient pathogen detection.

With the advancement of sequencing techniques, numerous genomes of *P. aeruginosa* and other *Pseudomonas* species have been described. Several novel specific target sequences, such as those of *gyrB*, *ecfX*, *fliC*, and *algD*, have been identified and applied to distinguish *P. aeruginosa* from other *Pseudomonas* spp. (Taee et al., 2014; Heidari et al., 2018; Wang et al., 2020; Khademi et al., 2021). The tremendous increase in the availability of bacterial genome sequences is allowing researchers to investigate and query pangenomes (Freschi et al., 2018).

Pangenome analysis has become a representative discipline for studying the entire repertoire of gene families in the genomes of pathogenic bacterial clades, which not only provides the whole set of genes shared by *Pseudomonas* species but also can also be applied in interspecies differentiation analysis to mine species-specific genes in order to use a wealth of genome data (Hilker et al., 2014).

**Figure 1:**
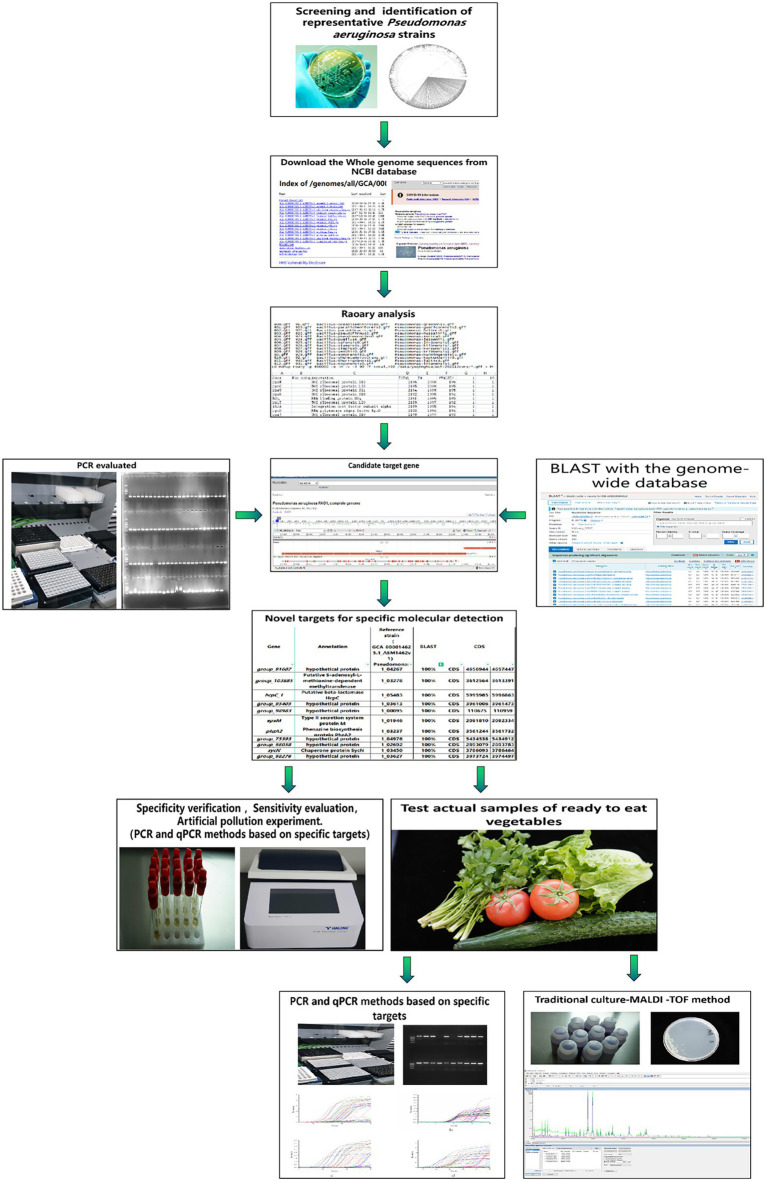
The flowchart of the experimental method involved in this study.

In short, for the detection of *P. aeruginosa*, traditional methods are time-consuming and laborious, and the experimental conditions of immunological methods are limited, while the sensitivity and accuracy of the existing molecular methods need to be considered. There is an urgent need for novel specific molecular detection targets of *P. aeruginosa* in order to establish a rapid and efficient detection method. Exactly, the explosive development of whole gene sequencing technology has made mining targets become convenient. Therefore, we aimed at mining novel specific target gene sequences of *P. aeruginosa* based on the pangenome analysis and established high-specificity and high-sensitivity PCR and quantitative real-time PCR (qPCR) methods based on these targets. Furthermore, the established methods were applied to the detection of actual samples of ready-to-eat vegetables to master the pollution of *P. aeruginosa* in ready-to-eat vegetable industry, so as to provide a scientific basis for reducing pollution. The flowchart of the experimental method involved in this study is shown in Figure 1.

## Materials and Methods

### Screening Species-Specific Novel Target Genes for *Pseudomonas aeruginosa*

Genomic sequences of 1,000 *P. aeruginosa* strains and 1,017 other common foodborne pathogen strains were retrieved from the NCBI Genome Database (last accessed on November 30, 2019). The specific information for the sequences is provided in Supplementary Table S1. Pangenome analysis was used to identify *P. aeruginosa* species-specific genes. The research involved the evaluation of nucleotide sequence dissimilarity between *P. aeruginosa* and non-*P. aeruginosa* sequences (Pang et al., 2019). In brief, all nucleic acid sequences downloaded from the NCBI database were annotated using Prokka v1.11 (Seemann, 2014). Then, the output of Prokka was used to construct a pangenome by Roary v3.11.2 (Page et al., 2015), with a BLASTP identity cutoff of 85%. The absence/existence profile of all genes across strains was converted into a 0/1 matrix with a local script. The matrix was then used to identify *P. aeruginosa* species-specific genes, which were screened according to the following criteria: 100% presence in target species strains and 0% presence in all other bacterial species strains and non-*P. aeruginosa* strains. Then, these candidate targets were further screened against the nucleotide collection (nr/nt) databases using the online BLAST program^1^ and PCR verification to ensure specificity.

### Specific Primer Design for PCR and Real-Time PCR

Primer Premier 6.0 software (PREMIER Biosoft International, Palo Alto, United States) was used to design primers targeting the screened conserved sequences of *P. aeruginosa*. Primers without hairpin structures or dimers and the highest rating score were selected. Their specificity was preliminarily verified by the NCBI Blast tool. Then, the primers listed in Table 1 were synthesized by Shanghai Sangon Company (Shanghai, China).

**Table 1:** Species-specific genes and primers for PCR and qPCR identification of *P. aeruginosa*.

Species	Name of target genes	^*^Gene location	Encoded protein	Primer set name	Sequences (5′-3′)	Product size (bp)	For PCR or qPCR assay
*P. aeruginosa*	*UCBPP-PA14_00095 (group_98983)*	110,675–110,959	Hypothetical protein	PA1	CTCCGTGGAAAAGCAGTTG	169	PCR
					GCGTATGCCGACGTAGAAT		
				PA12	AATGCGGGATGCTGCTCT	138	qPCR
					GGTCGGTCTCCTCGAACTCTT		
	*UCBPP-PA14_03237 (phzA2)*	3,561,244–3,561,732	Phenazine biosynthesis protein PhzA2	PA2	GTTTACCGACAACCTGGAA	325	PCR
					GCAATAGCCCTGCGGATAC		
				PA13	CAACTGGACCACGGAAAGC	126	qPCR
					GTCTCGAAGATCCGCACGT		
	*UCBPP-PA14_04976 (group_75393)*	5,434,538–5,434,912	Hypothetical protein	PA3	ATGGACAGGGACGCATTGA	263	PCR
					CGAGGGACGAAGGTAAGGA		
				PA14	CGGTACAGGTCGGCACG	109	qPCR
					CGAGGGACGAAGGTAAGGA		
	*UCBPP-PA14_03627 (group_88276)*	3,973,724–3,974,497	Hypothetical protein	PA4	GACTCTACCCTCCCTGACTT	132	PCR
					TCCATCACCGAGAAGC		
				PA15	TACGCGGTCAGCCATCAA	104	qPCR
					CAGCTCACTGCCGTTTCC		

* Reference strain is *P. aeruginosa* UCBPP-PA14. The reference gene is GCA_000014625.1_ASM1462v1.

### Bacterial Strains and Genomic DNA Extraction

This study used 134 bacterial strains (95 *P. aeruginosa* strains and 39 non-*P. aeruginosa* strains; Supplementary Table S2). They were purchased from the National Center for Medical Culture Collections (CMCC, Beijing, China), the American Type Culture Collection (ATCC, Manassas, VA, United States), and the China General Microbiological Culture Collection Center (CGMCC, Beijing, China). The other strains used in this study were part of our laboratory culture collection.

All strains were cultured in Luria-Bertani (LB) broth at 37°C. The bacterial cultures were then collected by centrifugation at 25°C and 12,000 × *g* for 5 min. Genomic DNA from these cells was extracted and purified using an EZNA Bacteria Genome Kit (Omega Bio-Tek Inc., Norcross, GA, United States) according to the manufacturer’s instructions. The concentration and purity of the DNA were estimated by agarose gel electrophoresis and by using a NanoDrop 2000c UV–Vis spectrophotometer (Thermo Fisher Scientific, Waltham, MA, United States). Extracted DNA was stored at −20°C until PCR and qPCR analysis.

### PCR and Real-Time PCR Conditions for *Pseudomonas aeruginosa* Detection

The DNA extracted from bacterial strains was used for PCR and qPCR amplification. The PCR mixture consisted of 12.5 μl of 2 × Taq Master Mix (Vazyme, China), 1 μl of each primer (10 μM), 50 ng of DNA template, and sterile distilled H_2_O up to a final volume of 25 μl. PCR amplification was performed in a PTC-100 programmable thermal controller (MJ Research, Inc.), with an initial denaturation step of 98°C for 3 min, followed by 35 cycles at 95°C for 30 s, 58.0°C for 30 s, and 72°C for 30 s and a final extension step at 72°C for 10 min. The PCR products were separated by 2% agarose gel electrophoresis and visualized by ethidium bromide staining. All PCR assays in this study were conducted in triplicate.

For qPCR amplification, the total reaction volume was 20 μl, including 10 μl of TB Green™ Premix Ex Taq™ II (TaKaRa, Biotech, Dalian, China), 1 μl each of the forward and reverse primers (10 μM), 7 μl of sterile water, and 50 ng of the purified bacterial genomic DNA as a template. A LightCycler® 96 System (Roche, Switzerland) was used for thermal cycling, as follows: initial denaturation of DNA at 95°C for 30 s, followed by 40 cycles of denaturation at 95°C for 5 s and annealing at 55°C for 60 s. The qPCR assay was performed in triplicate with parallel analysis in 96-well plates. Sterile water was used in place of the DNA template as a negative control to ensure the absence of contaminants.

### Specificity Evaluation of the Primers for PCR and qPCR Assays

All strains used for the verification of primer specificity in the PCR and qPCR assays were from our laboratory collection and are listed in Supplementary Table S2. Genomic DNA was extracted from 95 *P. aeruginosa* strains and 39 non-*P. aeruginosa* strains and used as a template to validate the specificity of the designed primers. One tube of PCR mixture was added to 2 μl of sterile distilled water instead of DNA template as a blank control. The PCR primer sets that could amplify a single target band with the expected length for the corresponding strains of *P. aeruginosa* that showed negative results for non-*P. aeruginosa* strains were considered species-specific primers and used for further evaluation. The reported *toxA* target gene, a major virulence factor in *P. aeruginosa*, was used in a comparative experiment (SN/T2206.12, 2016; Taee et al., 2014). The same experimental environment and strain sets and test set were maintained during the comparative experiment, only hanging the target to the *toxA* gene (Supplementary Table S4; Supplementary Figure S2).

Genomic DNAs from 63 *P. aeruginosa* strains and 32 other bacterial strains were used as a template for the qPCR amplification to evaluate the specificity of the qPCR assay. The qPCR assay was performed in triplicate with parallel analysis in 96-well plates (Supplementary Table S3).

### Sensitivity and Interference Evaluation of Specific Primers Using Genomic DNA

Purified DNA of a known concentration extracted from *P. aeruginosa* ATCC 15442 was serially diluted 10-fold. Two microliters of diluted extracted DNA was used as a template in a 25 μl PCR. One tube of PCR mixture was added to 2 μl of sterile distilled water instead of DNA template as a blank control. The PCR results were analyzed, and the detection limit of the PCR was determined. Then, 2 μl of each dilution was used as the template for qPCR amplification. A Light Cycler® 96 qPCR system (Roche, Basel, Switzerland) was used for thermal cycling as follows: denaturation at 95°C for 60 s, followed by 40 cycles of denaturation at 95°C for 10 s and annealing at 60°C for 30 s. The data were analyzed using built-in software. All *P. aeruginosa* DNA was extracted for qPCR analysis in triplicate. The target gene with the best detection limit was selected for further study.

*Pseudomonas aeruginosa* ATCC 15442 and a common pathogen (*Escherichia coli* ATCC 25922) were used to validate the PCR assay’s accuracy and scope for interference. The strains were cultured in LB broth at 37°C for 18 h and then serially diluted (10-fold) with 8.5% sodium chloride solution. The density of *P. aeruginosa* cells was adjusted to 10^4^ CFU/ml. *Pseudomonas aeruginosa* cultures were individually mixed with the interference testing strain at ratios of 1:10^3^, 1:10^2^, 1:10, 1:1, 10:1, and 10^2^:1, and 10^3^:1. Genomic DNA was extracted from the mixtures and used as a template for qPCR. Meanwhile, genomic DNA from *P. aeruginosa* cultures without the interference strain was used as the positive control template. The ability of the PCR assay to overcome interference was evaluated by 2.0% agarose gel electrophoresis.

### Artificial Contamination Experiments

*Pseudomonas aeruginosa* ATCC 15442 was cultured in LB broth at 37°C for 18 h, and the cell concentration was estimated by plate counting. Tomato samples (10 g) sterilized with ultraviolet light were mixed with 89 ml of LB medium, and then, the mixtures were incubated at 37°C for 18 h. Next, 1 ml *P. aeruginosa* mixtures were added at final inoculum concentrations ranging from 10^0^ to 10^8^ CFU/g. Genomic DNA was extracted at the indicated time points from 1 ml samples and then analyzed by PCR and qPCR. The amplification system and procedure were performed as described in “PCR and Real-Time PCR Conditions for *Pseudomonas aeruginosa* Detection” section.

### Detection of Pathogenic *Pseudomonas aeruginosa* in Samples of Ready-to-Eat Vegetables

A total of 29 ready-to-eat vegetable samples were collected from local markets in Guangdong Province, China, to validate the detection ability of PCR and qPCR. The ready-to-eat vegetables were sampled at random sites, and the samples were transported on ice to the laboratory for immediate analysis. The conventional culture method was used for testing based on the standard reference to detect *P. aeruginosa* in food for import and export (SN/T 2099-2008). Briefly, 25 g of each sample was randomly weighed, added to 225 ml of *P. aeruginosa* enrichment broth (SCDLP medium, Guangdong Huankai Co., Ltd., Guangzhou, China), and incubated at 37°C for 18 h. A loopful (approximately 10 μl) of the SCDLP enrichment culture was streaked into *P. aeruginosa-*selective agar plates (CN agar plates; Guangdong Huankai Co., Ltd., Guangzhou, China) and incubated at 37°C for 24 h. According to the manufacturer’s instructions, at least three presumptive colonies were selected to identify *P. aeruginosa* using the Bruker MALDI Biotyper identification system (MALDI, Bruker, Germany). Meanwhile, 1 ml of SCDLP broth enrichment culture was collected from each sample at 12 h. Genomic DNA was extracted from SCDLP broth enrichment cultures for PCR and qPCR.

## Results

### Identification of Specific Target Genes for *Pseudomonas aeruginosa*

Pangenome analysis was used to mine novel molecular targets for detecting *P. aeruginosa* in this study. A total of four genes (Table 1) were identified as specific to *P. aeruginosa* according to nucleotide sequence similarity. These gene sequences were present in 100% of the target *P. aeruginosa*, which did not exist in non-*P. aeruginosa* sequences available in the NCBI bacterial database according to BLASTN online.

After filtering using PCR analysis, four novel *P. aeruginosa*-specific targets, including *group_98983* (1,000/1,000), *phzA2* (1,000/1,000), *group_75393* (1,000/1,000), and *group_88276* (1,000/1,000), specific for the *P. aeruginosa* genes were uniquely present in all target strains but not in nontarget strains (Table 2; Supplementary Figure S1). The particular target gene *phzA2* encodes a phenazine biosynthesis protein, and the specific target genes *group_98983*, *group_75393*, and *group_88276* encode hypothetical proteins without assigned functions.

**Table 2:** Specificity results for PCR primers using *P. aeruginosa* and other foodborne pathogenic strains.

No.	Bacterial species	Strains	Number of strains	Source^*^	Species-specific target for PCR and qPCR							
					*PCR group_98983*	*PCR phzA2*	*PCR group_75393*	*PCR group_88276*	*qPCR group_98983*	*qPCR phzA2*	*qPCR group_75393*	*qPCR group_88276*
1	*P. aeruginosa*	^1^ATCC27853	1	a	+	+	+	+	+	+	+	+
2	*P. aeruginosa*	ATCC9027	1	a	+	+	+	+	+	+	+	+
3	*P. aeruginosa*	ATCC15442	1	a	+	+	+	+	+	+	+	+
4	*P. aeruginosa*	GIM1.46	1	b	+	+	+	+	+	+	+	+
5	*P. aeruginosa*	Laboratory isolate	91	a	+	+	+	+	(59)+	(59)+	(59)+	(59)+
6	*P. putida*	*ST25-10*	1	a	−	−	−	−	−	−	−	−
7	*P. putida*	*GIM1.57*	1	b	−	−	−	−	−	−	−	−
8	*P. fuscovaginae*	*ST42-2*	1	a	−	−	−	−	−	−	−	−
9	*P. hunanensis*	*0617-8*	1	a	−	−	−	−	−	−	−	−
10	*P. fulva*	*0625-4*	1	a	−	−	−	−	−	−	−	−
11	*P. kilonensis*	*ST38-5*	1	a	−	−	−	−	−	−	−	−
12	*P. lini*	*M41023-1*	1	a	−	−	−	−	−	−	−	−
13	*P. jessenii*	*ST42-4*	1	a	−	−	−	−	−	−	−	−
14	*P. alcaligenes*	^2^ *CMCC1.1806*	1	b	−	−	−	−	−	−	−	−
15	*P. chlororaphis*	*1,143-3*	1	a	−	−	−	−	−	−	−	−
16	*P. fragi*	*52,532-7*	1	a	−	−	−	−	−	−	−	−
17	*P. mendoza*	*CMCC1.1804*	1	b	−	−	−	−	−	−	−	−
18	*P. mosselii*	*ST42-10*	1	a	−	−	−	−	−	−	−	−
19	*P. corrugata*	*ST19-4*	1	a	−	−	−	−	−	−	−	−
20	*P. oleovorans*	*M43075-4*	1	a	−	−	−	−	−	−	−	−
21	*P. taiwanensis*	*0617-3*	1	a	−	−	−	−	−	−	−	−
22	*P. geniculata*	*52,023-3*	1	a	−	−	−	−	−	−	−	−
23	*P. fluorescens*	*51,184-3*	1	a	−	−	−	−	−	−	−	−
24	*P. fluorescens*	*GIM1.492*	1	b	−	−	−	−	−	−	−	−
25	*E. coli*	*ATCC 25922*	1	a	−	−	−	−	−	−	−	−
26	*E. coli*	*1,656-1*	1	a	−	−	−	−	−	−	−	−
27	*S. hominis*	*1,006-1*	1	a	−	−	−	−	−	−	−	−
28	*S. hominis*	*0656-4*	1	a	−	−	−	−	−	−	−	−
29	*S. haemolyticus*	*620*	1	a	−	−	−	−	−	−	−	−
30	*Y. enterocolitica*	*Y1408*	1	a	−	−	−	−	−	−	−	−
31	*Y. enterocolitica*	*C009*	1	a	−	−	−	−	−	−	−	−
32	*Y. enterocolitica*	*Y2602*	1	a	−	−	−	−	−	−	−	−
33	*Y. enterocolitica*	*Y3553*	1	a	−	−	−	−	−	−	−	−
34	*L. monocytogenes*	*1,333-2*	1	a	−	−	−	−	−	−	−	−
35	*L. monocytogenes*	*Feb-45*	1	a	−	−	−	−	−	−	−	−
36	*L. monocytogenes*	*509A1-3*	1	a	−	−	−	−	−	−	−	−
37	*E. coli*	*1,679*	1	a	−	−	−	−	−	−	−	−
38	*E. coli*	*1,677-3*	1	a	−	−	−	−	/	/	/	/
39	*S. epidermis*	*597*	1	a	−	−	−	−	/	/	/	/
40	*B. cereus*	*1,378*	1	a	−	−	−	−	/	/	/	/
41	*B. cereus*	*wqr5*	1	a	−	−	−	−	/	/	/	/
42	*S. aureus*	*800*	1	a	−	−	−	−	/	/	/	/
43	*Salmonella*	*839*	1	a	−	−	−	−	/	/	/	/
44	*Salmonella*	*838*	1	a	−	−	−	−	/	/	/	/

* a, our laboratory; b, Guangdong Huankai Co., Ltd., China. 1, ATCC, American Type Culture Collection, United States. 2, CMCC, China Medical Culture Collection, China. Results (+/−) indicate positive and negative signals.

### Diagnostic Specificity of the Novel Specific Primers

The results of specificity tests for the four PCR primer sets are shown in Table 2. These primers were prescreened with 95 *P. aeruginosa* strains and 39 non-*P. aeruginosa* strains. The four PCR primer sets showed perfect specificity for *P. aeruginosa*, and the bands of the four species-specific targets *group_98983*, *phzA2*, *group_75393*, and *group_88276* exhibited separate fragments of 169, 325, 263, and 132 bp, respectively, which were obtained only with *P. aeruginosa* as the template. All the non-*P. aeruginosa* strains displayed negative results. The above four novel genes had a coverage rate of 100% among existing genes in the strains, while the detection rate of *toxA* genes was only 82.1% (78/95; Supplementary Figure S2).

The sensitivity of the genes specific to *P. aeruginosa* DNA was further evaluated. We used qPCR for further analysis based on the specific primers screened by the PCR method. As shown in Table 2, we selected the PA12, PA13, PA14, and PA15 primer sets for use. For accurate qPCR analysis, four primer sets were designed (Table 1). A total of 63 *P. aeruginosa* strains and 32 non-*P. aeruginosa* strains were used to verify the specificity of the qPCR primers, and the results are shown in Table 2. According to the Ct values and dissolution curves, all non-*P. aeruginosa* strains showed no amplification, while amplification was obtained for the target *P. aeruginosa* strains, indicating a high specificity of the primers with qPCRs.

### Sensitivity Evaluation and Interference Evaluation of the Novel Specific Primers

**Figure 2:**
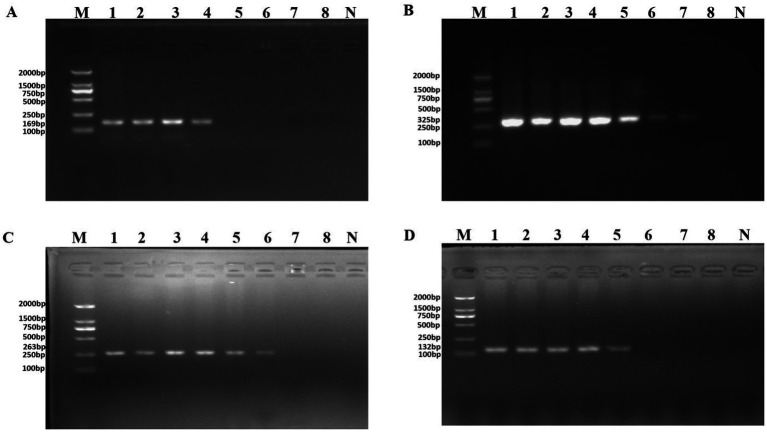
PCR detection sensitivity using dilutions of genomic DNA from *Pseudomonas aeruginosa* ATCC 15442. Lane M = DSTM 2000 marker (Dongsheng Biotechnology, Guangdong, China); lane N = negative control (double-distilled H_2_O); lanes 1–8 = 65.4 ng/μl, 6.54 ng/μl, 654 pg/μl, 65.4 pg/μl, 6.54 pg/μl, 654 fg/μl, and 65.4 fg/μl, 6.54 fg/μl, respectively. **(A)** Primer set PA1 (169 bp); **(B)** primer set PA2 (325 bp); **(C)** primer set PA3 (263 bp); and **(D)** primer set PA4 (132 bp).

The results regarding the specificity of the PCR assay with novel specific primers are shown in Supplementary Table S2. No product bands were obtained with the 39 non-*P. aeruginosa* strains tested, and no cross-reactivity was observed. To determine the detection limit of the novel assay, the initial concentration of DNA from *P. aeruginosa* ATCC 15442 was 65.4 ng/μl. The detection limits using the genomic DNA of *P. aeruginosa* with the PA1, PA2, PA3, and PA4 primer sets were 65.4 pg/μl, 65.4 fg/μl, 654 fg/μl, and 6.54 pg/μl, respectively (Figure 2).

**Figure 3:**
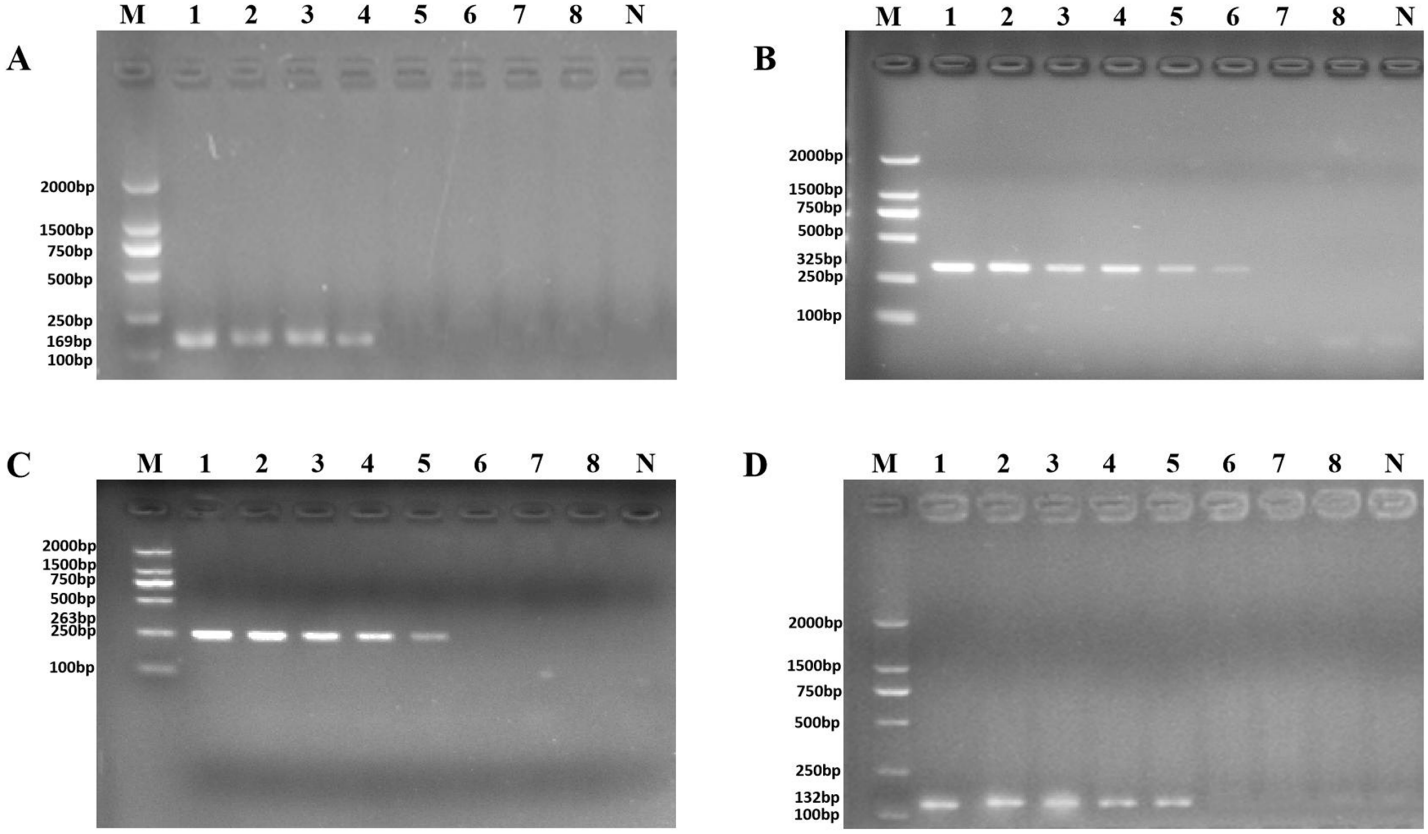
PCR detection sensitivity using dilutions of a pure culture of *P. aeruginosa* ATCC 15442. Lane M = DSTM 2000 marker (Dongsheng Biotechnology, Guangdong, China); lane N = negative control (double-distilled H_2_O); and lanes 1–8 = 2.07 × 10^8^ CFU/ml, 2.07 × 10^7^ CFU/ml, 1.85 × 10^6^ CFU/ml, 4.15 × 10^5^ CFU/ml, 4.3 × 10^4^ CFU/ml, 9.7 × 10^3^ CFU/ml, 1.4 × 10^2^ CFU/ml, and 2 × 10^1^ CFU/ml, respectively. **(A)** Primer set PA1 (169 bp); **(B)** primer set PA2 (325 bp); **(C)** primer set PA3 (263 bp); and **(D)** primer set PA4 (132 bp).

DNA was then extracted from different dilutions of *P. aeruginosa* cultures and used as the template. Following PCR detection, cell concentrations ranging from 10^0^ to 10^8^ CFU/ml were used. The detection limits observed whole cells of *P. aeruginosa* with the PA1, PA2, PA3, and PA4 primer sets were 4.15 × 10^5^ CFU/ml, 9.7 × 10^3^ CFU/ml, 4.3 × 10^4^ CFU/ml, and 4.3 × 10^4^ CFU/ml, respectively (Figure 3).

**Figure 4:**
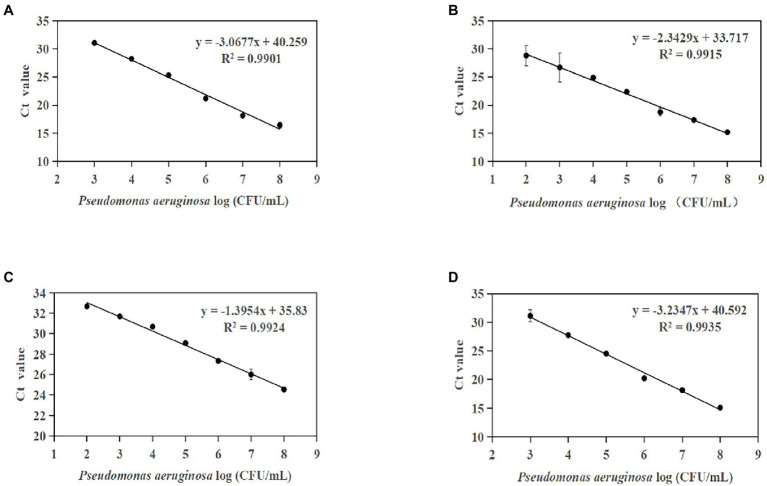
Standard curves established by plotting cycle threshold (Ct) values against the log numbers of *P. aeruginosa* in pure culture. **(A)** Primer set PA12 in a range of 10^3^–10^8^ CFU/ml; **(B)** primer set PA13 in a range of 10^2^–10^8^ CFU/ml; **(C)** primer set PA14 in a range of 10^2^–10^8^ CFU/ml; and **(D)** primer set PA15 in a range of 10^3^–10^8^ CFU/ml.

Standard curves were established based on the novel species-specific targets to quantify *P. aeruginosa*. As illustrated in Figures 4A–D, the four standard curves showed ideal linear correlations, with *R*^2^ values of 0.9901 for primer set PA12, 0.9915 for primer set PA13, 0.9924 for primer set PA14, and 0.9935 for primer set PA15. The detection limits were 10^3^ CFU/ml for primer sets PA12 and PA15 and 10^2^ CFU/ml for primer sets PA13 and PA14.

**Figure 5:**
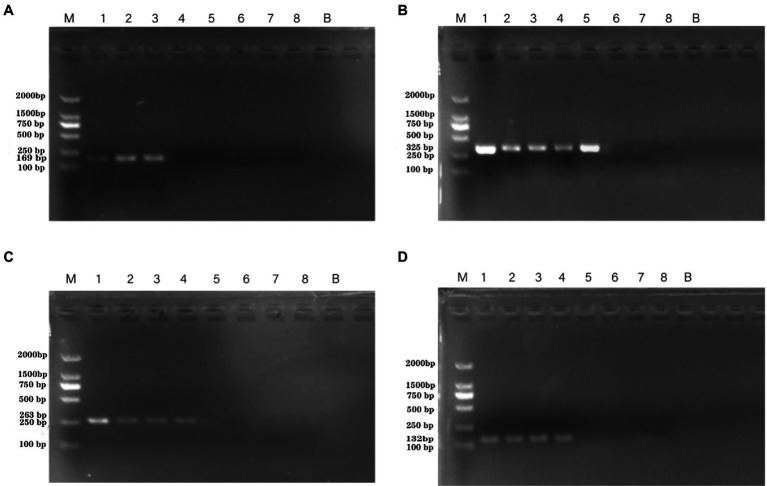
PCR detection sensitivity using dilutions of a pure culture of *P. aeruginosa* ATCC 15442 in spiked tomato lane M = DSTM 2000 marker (Dongsheng Biotechnology, Guangdong, China); lane N = negative control (double-distilled H_2_O); and lanes 1–8 = 1.33 × 10^8^ CFU/ml, 1.33 × 10^7^ CFU/ml, 1.33 × 10^6^ CFU/ml, 1.33 × 10^5^ CFU/ml, 1.33 × 10^4^ CFU/ml, 1.33 × 10^3^ CFU/ml, 1.33 × 10^2^ CFU/ml, and 2 × 10^1^ CFU/ml, respectively. **(A)** Primer set PA1 (169 bp); **(B)** primer set PA2 (325 bp); **(C)** primer set PA3 (263 bp); and **(D)** primer set PA4 (132 bp).

Artificially contaminated tomato was used to evaluate the sensitivity, specificity, and reliability of the primer sets PA1, PA2, PA3, and PA4. The cell concentrations of *P. aeruginosa* added to tomato were 10^1^–10^8^ CFU/ml. Following PCR detection, cell concentrations of 10^4^–10^6^ CFU/ml were used (Figure 5). The detection limits of the PA1, PA2, PA3, and PA4 primer sets were 1.33 × 10^6^ CFU/ml, 1.33 × 10^4^ CFU/ml, 1.33 × 10^5^ CFU/ml, and 1.33 × 10^5^ CFU/ml, respectively.

**Figure 6:**
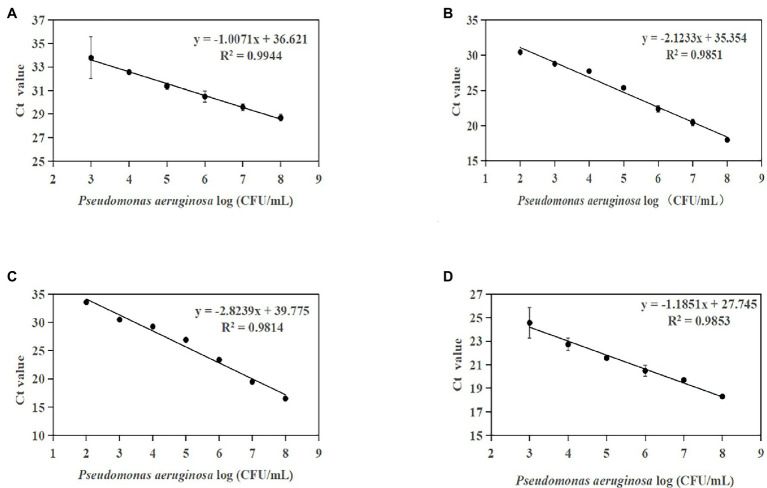
Standard curves established by plotting cycle threshold (Ct) values against the log numbers of *P. aeruginosa* in pure culture from artificially contaminated tomatoes. **(A)** Primer set PA12 in a range of 10^4^–10^8^ CFU/ml; **(B)** primer set PA13 in a range of 10^3^–10^8^ CFU/ml; **(C)** primer set PA14 in a range of 10^3^–10^8^ CFU/ml; and **(D)** primer set PA15 in a range of 10^4^–10^8^ CFU/ml.

Furthermore, the optimized conditions for the qPCR assay were used to establish a standard curve for *P. aeruginosa* detection in artificially contaminated samples. The linear detection range of these methods was 1.33 × 10^2^ CFU/g to 1.33 × 10^8^ CFU/g (Figures 6A–D). The four standard curves showed ideal linear correlations, with *R*^2^ values of 0.9944 for primer set PA12, 0.9851 for primer set PA13, 0.9814 for primer set PA14, and 0.9853 for primer set PA13. The LOD values of the four novel species-specific targets were 1.33 × 10^4^ CFU/g for primer sets PA12 and PA15, 1.33 × 10^3^ CFU/g for primer sets PA13 and PA14. Compared with the endpoint PCR method, the qPCR method was more sensitive by an order of magnitude.

**Figure 7:**
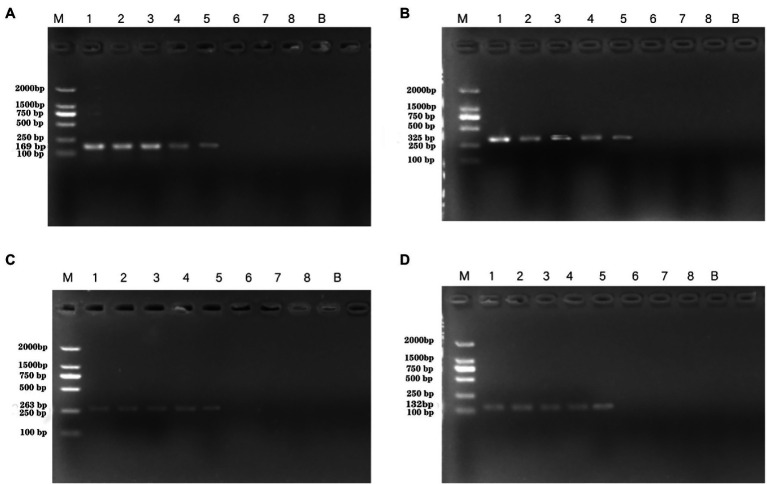
PCR assays using primers targeting *P. aeruginosa ATCC 15442* (4.6 × 10^4^ CFU/ml) testing for interference with different concentrations of *E. coli* ATCC 25922. Lane M = DSTM 2000 marker (Dongsheng Biotechnology, Guangdong, China); lanes 1–5 = mixtures of *Escherichia coli* ATCC 25922 concentration mixed with 1.462 × 10^8^ CFU/ml, 1.462 × 10^6^ CFU/ml, 1.462 × 10^4^ CFU/ml, 1.9 × 10^2^ CFU/ml, 0 CFU/ml, respectively. **(A)** Primer set PA1 (169 bp) mixed with *E. coli* ATCC 25922; **(B)** primer set PA2 (325 bp) mixed with *E. coli* ATCC 25922; **(C)** primer set PA3(263 bp) mixed with *E. coli* ATCC 25922; and **(D)** primer set PA4 (132 bp) mixed with *E. coli* ATCC 25922.

**Figure 8:**
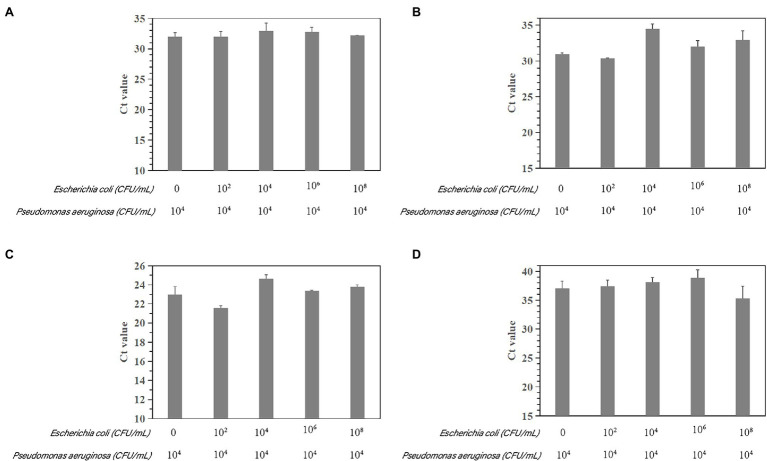
Assessment of interference with the real-time PCR-based detection of *P. aeruginosa* by coinfection with *E. coli* ATCC 25922. Detection of *P. aeruginosa* ATCC 15442 (4.6 × 10^4^ CFU/ml) in the presence of *E. coli* ATCC 25922 (0, 1.9 × 10^2^ CFU/ml, 1.462 × 10^4^ CFU/ml, 1.462 × 10^6^ CFU/ml, and 1.462 × 10^8^ CFU/ml). **(A)** Primer set PA12 mixed with *E. coli* ATCC 25922; **(B)** primer set PA13 mixed with *E. coli* ATCC 25922; **(C)** primer set PA14 mixed with *E. coli* ATCC 25922; and **(D)** primer set PA15 mixed with *E. coli* ATCC 25922.

The susceptibility of the PCR and qPCR assay to interference by nontarget DNA was determined by mixing *P. aeruginosa* and non-*P. aeruginosa* strains (*E. coli* ATCC 25922) at different ratios. Only one clear band was generated for mixtures of all strains tested for the PCR assay. The brightness of the band was comparable to that obtained by analyzing a pure *P. aeruginosa* culture (Figure 7). All amplifications had similar cycle threshold (Ct) values (Figure 8), regardless of the target-to-interfering strain ratio, suggesting that the presence of non-*P. aeruginosa* strains (*E. coli* ATCC 25922) did not interfere with *L. monocytogenes* serotype 4c detection. This result indicated that even a high abundance of *E. coli* ATCC 25922 did not interfere with the detection of *P. aeruginosa*.

### Application of the PCR Assay for the Analysis of Ready-to-Eat Vegetables

To verify the practicality and effectiveness of the developed PCR and qPCR methods, we next used these assays to detect *P. aeruginosa* in 29 unspiked ready-to-eat *vegetable* samples (Table 3). Among the 29 strains identified by the traditional MALDI (BRUKER, Germany) method, 14 ready-to-eat vegetable samples were positive. For species-specific targeting of *group_98983* and *group_88276* by the PCR and qPCR methods, the overall positive detection rate was 14/29, the same as that obtained with the traditional MALDI method. However, the PCR and qPCR methods with the species-specific target *phzA2* and *group_75393* were positive for 15 samples, consistent with the rate obtained by qPCRs. These results indicated that the four PCR primers and four qPCR primers designed by the novel species-specific target could be used to achieve the same positive detection results as the traditional MALDI method with the same initial inoculum. The established methods are accurate and reliable for the evaluation of actual samples of ready-to-eat vegetables.

**Table 3:** Test results for the detection of *P. aeruginosa* in ready-to-eat vegetable samples obtained using different methods.

Sample names	Sample types	Number of samples	Number of positive results obtained by different methods for *P. aeruginosa*								
			MALDI-TOF	PCR (*group_98983*)	PCR (*phzA2*)	PCR (*group_75393*)	PCR (*group_88276*)	PCR (*group_98983*)	qPCR (*phzA2*)	qPCR (*group_75393*)	qPCR (*group_88276*)
Ready-to-eat vegetables	Lettuce	7	2	2	3	3	2	2	3	3	2
	Coriander	7	4	4	4	4	4	4	4	4	4
	Tomatoes	8	5	5	5	5	5	5	5	5	5
	Cucumbers	7	3	3	3	3	3	3	3	3	3
	Total	29	14	14	15	15	14	14	15	15	14

## Discussion

The identification of *P. aeruginosa* has traditionally relied on phenotypic and biochemical methods, which take a long time to perform and require extensive hands-on work by the technologist, both for setup and for ongoing evaluation. Genotype-based identification methods circumvent the problem of variable phenotypes to enable more accurate species identification. Recently, molecular techniques have been developed for detecting *P. aeruginosa* based on its virulence genes, such as *toxA*, *ecfX*, *fliC*, and *oprL* (Taee et al., 2014; Wang et al., 2019, 2020).

However, deficiency and mutation of some virulence factors in *P. aeruginosa* strains can result in false results because of existing pathogenic factors, which may cause a potential threat of food poisoning (Baloyi et al., 2021). Since numerous microbial genome sequences have been completed and published with the development of sequencing technology and bioinformatics, many researchers have focused on exploring and screening novel specific target markers that could replace some target genes with poor specificity.

In this study, we developed PCR and qPCR methods to detect *P. aeruginosa* in food. The methods aimed at new species-specific gene targets were particular and sensitive. Vegetables from retail markets and supermarkets were widely contaminated by *P. aeruginosa* and have resistant or reduced susceptibilities antibiotic (Rahman et al., 2022). *Pseudomonas aeruginosa* as spoilage organisms in the ready-to-eat vegetables was distinguished by its capability to persist in highly antibiotic-resistant biofilm accumulation, which seriously affects shelf life (Allydice-Francis and Brown, 2012). While *P. aeruginosa* is considered an opportunistic pathogen, several reports have indicated that the organism can also cause infections in healthy hosts (Mateu-Borras et al., 2022). In addition, there was evidence that environmental isolates were as virulent as clinical strains (Li et al., 2018; D’Souza et al., 2020). Previous studies have found that *P. aeruginosa* can highly contaminate vegetables, revealing the potential hazard of salad vegetables and the possibility of food-related outbreaks of disease (Abrahale et al., 2019; Perez-Diaz et al., 2019; Villagran-de La Mora et al., 2020). Therefore, rapid detection of pathogenic *P. aeruginosa* is crucial in the vegetable supply chain. The consumption of ready-to-eat vegetables contaminated by *P. aeruginosa* may seriously impact human health.

However, traditional detection methods for *P. aeruginosa* may cause false positives or missed positives and are considerably time-consuming. Automated systems such as VITEK 2, which walkway system that works on the principle of photometry, promise shorter turnaround times to detect *P. aeruginosa*, but these systems have a low rate of accuracy in the identification (Torrecillas et al., 2020; Bhalla et al., 2021; Miranda-Ulloa et al., 2021; Pintado-Berninches et al., 2021; Viedma et al., 2021). Immunological approaches use the highly specific binding between antigens and antibodies and facilitate qualitative or quantitative detection that is based on specific reactions resulted from antigen antibody binding (Rainbow et al., 2020). High-sensitivity detection has been reached by modern immunoassay approaches, but their relatively tedious procedures have limited further development (Bhalla et al., 2021; Miranda-Ulloa et al., 2021; Viedma et al., 2021). In addition, greatest drawback of immunofluorescence methods is a low signal-to-noise ratio, which may lower its detection specificity (Pintado-Berninches et al., 2021). Electrochemical analysis can use the electrochemical characteristics of materials for qualitative and quantitative detection, which is fast and sensitive, but it needs compact experimental equipment to complete the experiment (Sabat et al., 2021; Zuccarello et al., 2021). Matrix-assisted laser desorption/ionization time of flight mass spectrometry (MALDI-TOF-MS) states an advanced technology and owns a very good application prospects in identifying *P. aeruginosa* (Wilhelm et al., 2021). MALDI-TOF-MS was used to accurately and rapidly identify the five high-risk clones of *P. aeruginosa* sequence ST111, ST175, ST235, ST253, and ST395, also was applied in *P. aeruginosa* drug resistance analysis such as carbapenemase (Mulet et al., 2021). MALDI-TOF-MS exhibits limited resolving power, and therefore does not supply sequence-based ID necessarily; microbial ID using MALDI-TOF-MS is based on spectral fingerprint patterns rather than the identity of each spectral peak (Ayhan et al., 2021).

The development of PCR-based detection methods for species-specific classification would provide an independent means for confirming species identity (Jami Al-Ahmadi and Zahmatkesh Roodsari, 2016). The current PCR detection methods for *P. aeruginosa* species target the virulence genes *toxA* or the 16S rRNA and 23S rRNA genes (Taee et al., 2014; Wang et al., 2016). With the development of sequencing technology and bioinformatics, many microbial genome sequences have been collected. Many researchers have sought to find new novel specific gene targets to replace the current target genes with poor specificity (Taee et al., 2014). Previously, specific target genes for *P. aeruginosa* was identified from sigma 70-factor sequences available from GenBank^2^ and then aligned using CLUSTALW software (Chowdhury and Garai, 2017; Wang et al., 2020). Neighbor-joining trees have been computed through the PHYLO_WIN graphical tool (Sánchez-Herrera et al., 2017). Specificity is the key to the success of conventional PCR, but it is also the most important reason for the failure of PCR detection. With the rapid development of whole-genome sequencing and bioinformatics, it has become more economical, convenient, and effective to identify specific targets by pangenome analysis than by using other molecular target screening methods. In this study, we used a large number of genome sequences (*n* = 2017) for pangenome analysis to identify specific gene targets of *P. aeruginosa*. According to the pangenome and PCR analyses, four novel *P. aeruginosa-*specific targets were 100% specific to the targeted *P. aeruginosa* genomes and did not detect nontarget *P. aeruginosa* genomes. However, the *P. aeruginosa*-specific targets reported in the previous studies, including *ecfX*, *16S rDNA*, *fliC*, *exotoxin A*, *oprI*, *algD*, and *oprL*, were present in 99.7%, 96.8%, 96.7%, 95%, 99.5%, 89.4%, and 96.9% of the target strains, respectively (Table 4). Except for the *fliC* gene, which showed low specificity, all of the genes had very high specificity, especially the *ecfX* and *gyrB* genes, whose detection was not associated with false positive or false negative results (Tang et al., 2017). In addition, the detection limits of primer pairs (10^3^–10^4^ CFU/ml) corresponding to these new target genes are similar to those of existing molecular detection targets (Tang et al., 2017). Consequently, the specific target of *P. aeruginosa* obtained by this method has good specificity. Its sensitivity can meet the needs of existing molecular detection methods. Moreover, it can represent the unique detection target of pathogenic *P. aeruginosa* in ready-to-eat vegetables and their downstream products.

**Table 4:** Presence profile of novel and reported *P. aeruginosa* species-specific gene targets for target and nontarget strains.

Species	Target genes	Presence profile in		Source
		Target strain	Nontarget strain	
*P. aeruginosa*	*group_98983*	1,000 (100%)	1,017 (0%)	This study
	*phzA2*	1,000 (100%)	1,017 (0%)	This study
	*group_75393*	1,000 (100%)	1,017 (0%)	This study
	*group_88276*	1,000 (100%)	1,017 (0%)	This study
	*ecfX*	1,000 (99.7%)	1,017 (1.4%)	Wang et al., 2020
	*16S rDNA*	1,000 (96.8%)	1,017 (1.4%)	Wang et al., 2016
	*fliC*	1,000 (96.7%)	1,017 (1.4%)	Ertugrul et al., 2018
	*toxA*	1,000 (95%)	1,017 (1%)	Ertugrul et al., 2018
	*oprL*	1,000 (99.5%)	1,017 (0.9%)	Taee et al., 2014
	*algD*	1,000 (89.4%)	1,017 (0.9%)	Heidari et al., 2018
	*oprI*	1,000 (96.9%)	1,017 (0.8%)	Mapipa et al., 2021

The PCR assay developed in the current study combines four specific primer sets (PA1, PA2, PA3, and PA4) based on novel molecular markers (*UCBPP-PA14_00095*, *UCBPP-PA14_03237*, *UCBPP-PA14_04976*, and *UCBPP-PA14_03627*, respectively) and allows simultaneous identification of pathogenic *P. aeruginosa*. The minimum detection limits of the assays were 10^3^–10^4^ CFU/ml for *P. aeruginosa* when pure enriched cultures were analyzed, which are comparable to those for PCRs reported in previous studies (Tang et al., 2017). These observations indicated that the new PCR assay could be used to detect *P. aeruginosa* in samples more rapidly (the overall assay time, including 4–12 h of pre-enrichment, DNA extraction, and the PCR assay, was only 5–17 h) than by using the standard culture method (4–7 days).

We designed the primers PA1, PA2, PA3, and PA4 according to the targets *UCBPP-PA14_00095*, *UCBPP-PA14_03237*, *UCBPP-PA14_04976*, and *UCBPP-PA14_03627*, respectively. Real-time PCR methods were established on the basis of the above findings. The minimum detection limit of the qPCR assay for *P. aeruginosa* was 10^2^ CFU/ml. The equations of the qPCR method showed good linearity. These values, comparable to those of most qPCR methods used for foods, were obtained without prior enrichment. Sarabaegi and Roushani (2019) reported a qPCR assay that detected a level of 2.7 × 10^2^ CFU/ml for *P. aeruginosa* in water.

Similarly, Fortunato et al. (2021) used a qPCR method to detect *P. aeruginosa* in soil and manure with a detection limit of 10^4^ CFU/g. Notably, the entire assay, including DNA extraction and qPCR, can be completed within 2 h. Compared with other assays, such as traditional culture and conventional PCR methods, the qPCR assay is more sensitive, more specific, time-efficient, and labor-saving.

We applied these methods to detect *P. aeruginosa* in actual samples of ready-to-eat vegetables, the results of which were consistent with the results of traditional culture methods. The positivity rate of *P. aeruginosa* was approximately 50% (*n* = 29), which was equivalent to that for fresh-cut fruits and vegetables (Savic et al., 2021). The positivity rate showed that the contamination of ready-to-eat vegetables by *P. aeruginosa* was significantly higher than that of other types of food, such as cooked meat products, cold ready-to-eat foods, and drinking water which was 6.25%, 17.65%, and 1.19%, respectively (Cai et al., 2015). This favorable rate of *P. aeruginosa* was due to dominant flora of vegetable plant saprophytic bacteria (Jin et al., 2021). *Pseudomonas aeruginosa* carried by water sources and raw materials may also cause contamination at all points in the sequence of vegetable irrigation, circulation, and clean vegetable processing in an open environment and eventually contaminate ready-to-eat vegetable products. It was found that *P. aeruginosa* strains from vegetables were genetically and functionally similar to clinical isolates in genetics and function (Ambreetha et al., 2021). Therefore, the development of rapid and sensitive detection methods for *P. aeruginosa* in ready-to-eat vegetables is of great significance to epidemiological research.

In this research, the methods of basic new specific molecular targets face two major limitations in practical application. On the one hand, with the increase of the number of test samples, false positive results may occur. On the other hand, this method belongs to the variable temperature nucleic acid amplification method, which needs to use a specific variable temperature amplification instrument to successfully complete the experiment. Although the method based on new specific targets can grasp the pollution level of *P. aeruginosa* in ready-to-eat vegetables, there are many uncertainties in the transmission mechanism of *P. aeruginosa* in the food chain. In subsequent experiments, we plan to analyze the serotype and evolutionary relationship of *P. aeruginosa* strains isolated from food samples in order to trace the way of *P. aeruginosa* contamination.

## Conclusion

In conclusion, we successfully mined four novel specific target gene sequences of *P. aeruginosa* with high specificity and sensitivity used pangenome analysis. Based on these new targets, high-specificity and high-sensitivity PCR and qPCR assays were established for rapid detection of *P. aeruginosa*. Furthermore, the established PCR and qPCR methods were applied to the whole cell detection in practical samples of ready-to-eat vegetables. Comparing the positive results of *P. aeruginosa* in ready-to-eat vegetables, the detection method based on the new target is consistent with the detection method of standard culture and is not disturbed by nontarget bacteria in the detection environment. Hence, the developed assays based on the novel specific target can be applied for rapid screening and detecting *P. aeruginosa* in ready-to-eat vegetables, providing a scientific basis for the monitoring of foodborne *P. aeruginosa*.

## Data Availability Statement

The original contributions presented in the study are included in the article/Supplementary Material; further inquiries can be directed to the corresponding authors.

## Author Contributions

CW: investigation, methodology, data curation, and writing original draft. QY: project administration and data curation. AJ and JZ: supervision and resources. YS, FL, BZ, XX, QG, RP, and YD: data curation. SW and MC: validation. QW and JW: supervision and writing review and editing. All authors contributed to the article and approved the submitted version.

## Funding

This study was supported by National Key Research and Development Program of China (2017YFC1600403), Guangdong Provincial Key Laboratory (2020B121201009) and GDAS’ Special Project of Science and Technology Development (2020GDASYL-20200401002).

## Conflict of Interest

The authors declare that the research was conducted in the absence of any commercial or financial relationships that could be construed as a potential conflict of interest.

## Publisher’s Note

All claims expressed in this article are solely those of the authors and do not necessarily represent those of their affiliated organizations, or those of the publisher, the editors and the reviewers. Any product that may be evaluated in this article, or claim that may be made by its manufacturer, is not guaranteed or endorsed by the publisher.

## Footnotes

^1^ https://blast.ncbi.nlm.nih.gov/Blast.cgi

^2^ https://www.ncbi.nlm.nih.gov/

## References

[ref1] Abrahale K. Sousa S. Albuquerque G. Padrao P. Lunet N. (2019). Street food research worldwide: a scoping review. J. Hum. Nutr. Diet. 32, 152–174. doi: 10.1111/jhn.12604, PMID: 30311276

[ref2] Allydice-Francis K. Brown P. D. (2012). Diversity of antimicrobial resistance and virulence determinants in *Pseudomonas aeruginosa* associated with fresh vegetables. Int. J. Microbiol. 2012:426241. doi: 10.1155/2012/426241 23213336 PMC3508576

[ref3] Ambreetha S. Marimuthu P. Mathee K. Balachandar D. (2021). Rhizospheric and endophytic *Pseudomonas aeruginosa* in edible vegetable plants share molecular and metabolic traits with clinical isolates. J. Appl. Microbiol. doi: 10.1111/jam.15317, PMID: 34608722

[ref4] Ayhan K. Cosansu S. Orhan-Yanikan E. Gulseren G. (2021). Advance methods for the qualitative and quantitative determination of microorganisms. Microchem. J. 166:106188, 106188. doi: 10.1016/j.microc.2021.106188

[ref5] Baloyi I. T. Adeosun I. J. Yusuf A. A. Cosa S. (2021). In silico and in vitro screening of antipathogenic properties of *Melianthus comosus* (Vahl) against *Pseudomonas aeruginosa*. Antibiotics 10, 1–23. doi: 10.3390/antibiotics10060679 PMC8230066 34198845

[ref6] Bhalla M. Heinzinger L. R. Morenikeji O. B. Marzullo B. Thomas B. N. Ghanem E. N. B. (2021). Transcriptome profiling reveals CD73 and age-driven changes in neutrophil responses against *Streptococcus pneumoniae*. Infect. Immun. 89:e0025821. doi: 10.1128/IAI.00258-21, PMID: 34310891 PMC8519284

[ref7] Cai S. F. Zhang Q. Huang Y. X. (2015). Monitoring and analysis of *Pseudomonas aeruginosa* in food. Chin. J. Health Lab. Technol. 26, 875–905.

[ref8] Chon J. Jung J. Y. Ahn Y. Bae D. Khan S. Seo K.. (2021). Detection of campylobacter jejuni from fresh produce: comparison of culture- and PCR-based techniques, and metagenomic approach for analyses of the microbiome before and after enrichment. J. Food Prot. 84, 1704–1712. doi: 10.4315/JFP-20-408, PMID: 33878155

[ref9] Chowdhury B. Garai G. (2017). A review on multiple sequence alignment from the perspective of genetic algorithm. Genomics 109, 419–431. doi: 10.1016/j.ygeno.2017.06.007, PMID: 28669847

[ref10] Crull M. R. Somayaji R. Ramos K. J. Caldwell E. Mayer-Hamblett N. Aitken M. L.. (2018). Changing rates of chronic *Pseudomonas aeruginosa* infections in cystic fibrosis: a population-based cohort study. Clin. Infect. Dis. 67, 1089–1095. doi: 10.1093/cid/ciy215, PMID: 29534149 PMC6137120

[ref11] Dharmarha V. Guron G. Boyer R. R. Niemira B. A. Pruden A. Strawn L. K.. (2019). Gamma irradiation influences the survival and regrowth of antibiotic-resistant bacteria and antibiotic-resistance genes on romaine lettuce. Front. Microbiol. 10:710. doi: 10.3389/fmicb.2019.00710, PMID: 31024491 PMC6465624

[ref12] D'Souza C. Prithvisagar K. S. Deekshit V. K. Karunasagar I. Kumar B. K. (2020). Exploring the pathogenic potential of *Vibrio vulnificus* isolated from seafood harvested along the Mangaluru coast, India. Microorganisms 8:999. doi: 10.3390/microorganisms8070999, PMID: 32635463 PMC7409051

[ref300] Ertugrul B. M. Oryasin E. Lipsky B. A. Willke A. Bozdogan B. (2018). Virulence genes fliC, toxA and phzS are common among Pseudomonas aeruginosa isolates from diabetic foot infections. Infect. Dis. (Lond). 50, 273–279. doi: 10.1080/23744235.2017.1393839, PMID: 29078729

[ref13] Fakhkhari P. Tajeddin E. Azimirad M. Salmanzadeh-Ahrabi S. Abdi-Ali A. Nikmanesh B.. (2022). Involvement of *Pseudomonas aeruginosa* in the occurrence of community and hospital acquired diarrhea, and its virulence diversity among the stool and the environmental samples. Int. J. Environ. Health Res. 32, 61–71. doi: 10.1080/09603123.2020.1726300, PMID: 32073302

[ref14] Fortunato G. Vaz-Moreira I. Nunes O. C. Manaia C. M. (2021). Effect of copper and zinc as sulfate or nitrate salts on soil microbiome dynamics and blaVIM-positive *Pseudomonas aeruginosa* survival. J. Hazard. Mater. 415:125631. doi: 10.1016/j.jhazmat.2021.125631, PMID: 33773246

[ref15] Freschi L. Vincent A. T. Jeukens J. Emond-Rheault J. G. Kukavica-Ibrulj I. Dupont M.. (2018). The *Pseudomonas aeruginosa* pan-genome provides new insights on its population structure, horizontal gene transfer, and pathogenicity. Genome Biol. Evol. 11, 109–120. doi: 10.1093/gbe/evy259, PMID: 30496396 PMC6328365

[ref16] Gharieb R. Saad M. Khedr M. El Gohary A. Ibrahim H. (2022). Occurrence, virulence, carbapenem resistance, susceptibility to disinfectants and public health hazard of *Pseudomonas aeruginosa* isolated from animals, humans and environment in intensive farms. J. Appl. Microbiol. 132, 256–267. doi: 10.1111/jam.15191, PMID: 34171153

[ref17] Godova G. V. Ovod A. A. Astakhova N. V. Kalashnikova Y. A. (2020). Histological study of lettuce and basil by infection with *Ps. aeruginosa* and *Ps. fluorescens* in vitro. Izv. Timi. Agri. Acad. 56–69. doi: 10.26897/0021-342X-2020-3-56-69

[ref18] Heidari H. Hadadi M. Sedigh Ebrahim-Saraie H. Mirzaei A. Taji A. Hosseini S. R.. (2018). Characterization of virulence factors, antimicrobial resistance patterns and biofilm formation of *Pseudomonas aeruginosa* and *Staphylococcus* spp. strains isolated from corneal infection. J. Fr. Ophtalmol. 41, 823–829. doi: 10.1016/j.jfo.2018.01.012, PMID: 30292385

[ref19] Hilker R. Munder A. Klockgether J. Losada P. M. Tümmler B. (2014). Interclonal gradient of virulence in the *Pseudomonas aeruginosa* pangenome from disease and environment. Environ. Microbiol. 17, 29–46. doi: 10.1111/1462-2920.12606 25156090

[ref20] Hölzel C. S. Tetens J. L. Schwaiger K. (2018). Unraveling the role of vegetables in spreading antimicrobial-resistant bacteria: a need for quantitative risk assessment. Foodborne Pathog. Dis. 15, 671–688. doi: 10.1089/fpd.2018.2501, PMID: 30444697 PMC6247988

[ref21] Jami Al-Ahmadi G. Zahmatkesh Roodsari R. (2016). Fast and specific detection of *Pseudomonas aeruginosa* from other *pseudomonas* species by PCR. Ann. Burn. Fire Disasters 29, 264–267. PMC5347312 28289359

[ref22] Jin S. Ding Z. Xie J. (2021). Study of postharvest quality and antioxidant capacity of freshly cut amaranth after blue LED light treatment. Plants 10:1614. doi: 10.3390/plants10081614, PMID: 34451660 PMC8400882

[ref23] Junaid K. Ejaz H. Asim I. Younas S. Yasmeen H. Abdalla A. E.. (2021). Heavy metal tolerance trend in extended-spectrum beta-lactamase encoding strains recovered from food samples. Int. J. Environ. Res. Public Health 18:4718. doi: 10.3390/ijerph18094718, PMID: 33925201 PMC8124721

[ref24] Kapeleka J. A. Sauli E. Sadik O. Ndakidemi P. A. (2020). Co-exposure risks of pesticides residues and bacterial contamination in fresh fruits and vegetables under smallholder horticultural production systems in Tanzania. PLoS One 15:e0235345. doi: 10.1371/journal.pone.0235345, PMID: 32667930 PMC7363064

[ref25] Khademi F. Maarofi K. Arzanlou M. Dogaheh H. P. Sahebkar A. (2021). Which missense mutations associated with DNA gyrase and topoisomerase IV are involved in *Pseudomonas aeruginosa* clinical isolates resistance to ciprofloxacin in Ardabil? Gene Rep. 24:101211. doi: 10.1016/j.genrep.2021.101211

[ref26] Kidd T. J. Magalhaes R. J. S. Paynter S. Bell S. C. (2015). The social network of cystic fibrosis centre care and shared *Pseudomonas aeruginosa* strain infection: a cross-sectional analysis. Lancet Respir. Med. 3, 640–650. doi: 10.1016/S2213-2600(15)00228-3, PMID: 26208994

[ref27] Kwok W. C. Ho J. C. M. Tam T. C. C. Ip M. S. M. Lam D. C. L. (2021). Risk factors for *Pseudomonas aeruginosa* colonization in non-cystic fibrosis bronchiectasis and clinical implications. Respir. Res. 22:132. doi: 10.1186/s12931-021-01729-5, PMID: 33910573 PMC8080398

[ref28] Li Y. Liu X. Tang K. Wang P. Zeng Z. Guo Y.. (2018). Excisionase in Pf filamentous prophage controls lysis-lysogeny decision-making in *Pseudomonas aeruginosa*. Mol. Microbiol. 111, 495–513. doi: 10.1111/mmi.14170, PMID: 30475408 PMC7379572

[ref29] Mapipa Q. Digban T. O. Nnolim N. E. Nwodo U. U. (2021). Antibiogram profile and virulence signatures of *Pseudomonas aeruginosa* isolates recovered from selected agrestic hospital effluents. Sci. Rep. 11:11800. doi: 10.1038/s41598-021-91280-6, PMID: 34083705 PMC8175747

[ref30] Maske B. L. Pereira G. V. D. M. Neto D. P. D. C. Lindner J. D. D. Letti L. A. J. Pagnoncelli M. G.. (2021). Presence and persistence of *Pseudomonas sp.* during Caspian Sea-style spontaneous milk fermentation highlights the importance of safety and regulatory concerns for traditional and ethnic foods. Food Sci. Technol. 41, 273–283. doi: 10.1590/fst.15620

[ref31] Mateu-Borras M. Zamorano L. Gonzalez-Alsina A. Sanchez-Diener I. Domenech-Sanchez A. Oliver A.. (2022). Molecular analysis of the contribution of alkaline protease A and elastase B to the virulence of *Pseudomonas aeruginosa* bloodstream infections. Front. Cell. Infect. Microbiol. 11:816356. doi: 10.3389/fcimb.2021.816356, PMID: 35145924 PMC8823171

[ref32] Mesinele J. Ruffin M. Kemgang A. Guillot L. Boelle P. Corvol H. (2022). Risk factors for *Pseudomonas aeruginosa* airway infection and lung function decline in children with cystic fibrosis. J. Cyst. Fibros. 21, 45–51. doi: 10.1016/j.jcf.2021.09.017, PMID: 34629287

[ref33] Miranda-Ulloa E. Romero-Ruiz S. Amorín-Uscata B. Serrano-Segura K. Briceño-Espinoza R. Cárdenas-Bustamante F. (2021). Estandarización y validación de un Western Blot para el diagnóstico del virus de inmunodeficiencia humana. Rev. Fac. Med. Hum. 21, 674–681. doi: 10.25176/rfmh.v21i4.4023

[ref34] Mulet X. Fernandez-Esgueva M. Norte C. Zamorano L. Del Barrio-Tofino E. Oliver A. (2021). Validation of MALDI-TOF for the early detection of the ST175 high-risk clone of *Pseudomonas aeruginosa* in clinical isolates belonging to a Spanish nationwide multicenter study. Enferm. Infecc. Microbiol. Clin. 39, 279–282. doi: 10.1016/j.eimc.2020.05.022, PMID: 34088448

[ref35] Namaki M. Habibzadeh S. Vaez H. Arzanlou M. Safarirad S. Bazghandi S. A.. (2022). Prevalence of resistance genes to biocides in antibiotic-resistant *Pseudomonas aeruginosa* clinical isolates. Mol. Biol. Rep. 49, 2149–2155. doi: 10.1007/s11033-021-07032-2, PMID: 34854015

[ref36] Naze F. Jouen E. Randriamahazo R. T. Simac C. Laurent P. Bleriot A.. (2010). *Pseudomonas aeruginosa* outbreak linked to mineral water bottles in a neonatal intensive care unit: fast typing by use of high-resolution melting analysis of a variable-number tandem-repeat locus. J. Clin. Microbiol. 48, 3146–3152. doi: 10.1128/JCM.00402-10, PMID: 20573865 PMC2937704

[ref200] Oliver A. Mulet X. Lopez-Causape C. Juan C. (2015). The increasing threat of Pseudomonas aeruginosa high-risk clones. Drug Resistance Updates 21–22, 41–59. doi: 10.1016/j.drup.2015.08.002, PMID: 26304792

[ref37] Page A. J. Cummins C. A. Martin H. Wong V. K. Sandra R. Holden M.. (2015). Roary: rapid large-scale prokaryote pan genome analysis. Bioinformatics 31, 3691–3693. doi: 10.1093/bioinformatics/btv421, PMID: 26198102 PMC4817141

[ref38] Pang R. Xie T. Wu Q. Li Y. Lei T. Zhang J.. (2019). Comparative genomic analysis reveals the potential risk of vibrio parahaemolyticus isolated from ready-to-eat foods in China. Front. Microbiol. 10:186. doi: 10.3389/fmicb.2019.00186 30792709 PMC6374323

[ref39] Pelegrin A. C. Palmieri M. Mirande C. Oliver A. Moons P. Goossens H.. (2021). *Pseudomonas aeruginosa*: a clinical and genomics update. FEMS Microbiol. Rev. 45:fuab026. doi: 10.1093/femsre/fuab026, PMID: 33970247

[ref40] Perez-Diaz I. M. Hayes J. S. Medina E. Webber A. M. Butz N. Dickey A. N.. (2019). Assessment of the non-lactic acid bacteria microbiota in fresh cucumbers and commercially fermented cucumber pickles brined with 6% NaCl. Food Microbiol. 77, 10–20. doi: 10.1016/j.fm.2018.08.003, PMID: 30297040

[ref41] Pintado-Berninches L. Montes-Worboys A. Manguan-Garcia C. Arias-Salgado E. G. Serrano A. Fernandez-Varas B.. (2021). GSE4-loaded nanoparticles a potential therapy for lung fibrosis that enhances pneumocyte growth, reduces apoptosis and DNA damage. FASEB J. 35:e21422. doi: 10.1096/fj.202001160RR, PMID: 33638895 PMC12266330

[ref42] Rahman M. Alam M. Luies S. K. Kamal A. Ferdous S. Lin A.. (2022). Contamination of fresh produce with antibiotic-resistant bacteria and associated risks to human health: a scoping review. Int. J. Environ. Res. Public Health 19:360. doi: 10.3390/ijerph19010360, PMID: 35010620 PMC8744955

[ref43] Rainbow J. Sedlackova E. Jiang S. Maxted G. Moschou D. Richtera L.. (2020). Integrated electrochemical biosensors for detection of waterborne pathogens in low-resource settings. Biosensors 10:36. doi: 10.3390/bios10040036, PMID: 32294961 PMC7236604

[ref44] Ruiz-Roldán L. Rojo-Bezares B. Lozano C. López M. Chichón G. Torres C.. (2021). Occurrence of *Pseudomonas* spp. in raw vegetables: molecular and phenotypical analysis of their antimicrobial resistance and virulence-related traits. Int. J. Mol. Sci. 22:12626. doi: 10.3390/ijms222312626, PMID: 34884433 PMC8657893

[ref45] Sabat A. J. Pantano D. Akkerboom V. Bathoorn E. Friedrich A. W. (2021). *Pseudomonas aeruginosa* and *Staphylococcus aureus* virulence factors as biomarkers of infection. Biol. Chem. 402, 1565–1573. doi: 10.1515/hsz-2021-0243, PMID: 34505460

[ref46] Sánchez-Herrera K. Sandoval H. Mouniee D. Ramírez-Durán N. Bergeron E. Boiron P.. (2017). Molecular identification of *Nocardia* species using the sodA gene: identificación molecular de especies de *Nocardia* utilizando el gen sodA. New Microbes New Infect. 19, 96–116. doi: 10.1016/j.nmni.2017.03.008, PMID: 28794885 PMC5547243

[ref47] Sarabaegi M. Roushani M. (2019). A nano-sized chitosan particle based electrochemical aptasensor for sensitive detection of *P. aeruginosa*. Anal. Methods 11, 5591–5597. doi: 10.1039/c9ay01509d

[ref48] Savic A. Topalic-Trivunovic L. Velemir A. Papuga S. Kalaba V. (2021). Attachment and survival of bacteria on apples with the creation of a kinetic mathematical model. Braz. J. Microbiol. 52, 837–846. doi: 10.1007/s42770-021-00425-2, PMID: 33484470 PMC8105475

[ref49] Schroth M. N. Cho J. J. Green S. K. Kominos S. D. (2018). Epidemiology of *Pseudomonas aeruginosa* in agricultural areas. J. Med. Microbiol. 67, 1191–1201. doi: 10.1099/jmm.0.000758, PMID: 30067169

[ref50] Seemann T. (2014). Prokka: rapid prokaryotic genome annotation. Bioinformatics 30, 2068–2069. doi: 10.1093/bioinformatics/btu153, PMID: 24642063

[ref51] Taee S. R. Khansarinejad B. Abtahi H. Najafimosleh M. Ghaznavi-Rad E. (2014). Detection of algD, oprL and exoA genes by new specific primers as an efficient, rapid and accurate procedure for direct diagnosis of *Pseudomonas aeruginosa* strains in clinical samples. Jundishapur J. Microbiol. 7:e13583. doi: 10.5812/jjm.13583, PMID: 25632330 PMC4295320

[ref52] Tang Y. Ali Z. Zou J. Jin G. Zhu J. Yang J.. (2017). Detection methods for *Pseudomonas aeruginosa*: history and future perspective. RSC Adv. 7, 51789–51800. doi: 10.1039/C7RA09064A

[ref53] Torrecillas M. Fuster B. Belda M. Del Remedio Guna M. Tormo N. Gimeno C. (2020). Evaluation of a mass spectrometry and Vitek 2 combined protocol for rapid identification and susceptibility testing of enterobacterales directly from positive blood cultures. Enferm. Infecc. Microbiol. Clin. 38, 375–378. doi: 10.1016/j.eimc.2019.12.012, PMID: 32057553

[ref54] Tungpradabkul S. Senapin S. Panyim S. (2005). PCR-based method for isolation of the flagellin genes from *Pseudomonas species*. J. Gen. Appl. Microbiol. 44, 231–234. doi: 10.1016/S0277-5387(97)00461-0 12501433

[ref55] Viedma M. D. P. M. Panossian S. Gifford K. Garcia K. Figueroa I. Parham L.. (2021). Evaluation of ELISA-based multiplex peptides for the detection of human serum antibodies induced by Zika virus infection across various countries. Viruses 13:1319. doi: 10.3390/v13071319, PMID: 34372525 PMC8310037

[ref56] Villagran-de La Mora Z. Esther Macias-Rodriguez M. Arratia-Quijada J. Sughey Gonzalez-Torres Y. Nuno K. Villarruel-Lopez A. (2020). Clostridium perfringens as foodborne pathogen in broiler production: pathophysiology and potential strategies for controlling necrotic enteritis. Animals 10:1718. doi: 10.3390/ani10091718, PMID: 32972009 PMC7552638

[ref57] Wang Y. Geng Y. Hao B. (2016). Study on the detection method of Rhodopseudomonas palustris with 16S rDNA PCR. Sichuan Animal & Veterinary Sciences. 5, 20–25.

[ref58] Wang J. Xiang J. Sun X. Li R. Jiang Y. Chen Z.. (2020). Development and evaluation of a real-time recombinase-aid amplification assay for rapid detection of *Pseudomonas aeruginosa*. Chin. J. Food Hygiene 32, 524–529. doi: 10.13590/j.cjfh.2020.05.010

[ref59] Wang Z. Zuo J. Gong J. Hu J. Han X. (2019). Development of a multiplex PCR assay for the simultaneous and rapid detection of six pathogenic bacteria in poultry. AMB Express 9:185. doi: 10.1186/s13568-019-0908-0, PMID: 31728678 PMC6856251

[ref60] Wei L. Qing-Ping W. U. Zhang J. M. Ke-Gang W. U. Guo W. P. Que S. H. (2015). The pollution survey of *Pseudomonas aeruginosa* in mineral water and spring water and the analyses of virulence genes and antibiotic resistance of the isolates. Microbiol. China 42, 125–132. doi: 10.13344/j.microbiol.china.140331

[ref61] Wilhelm C. M. Forni G. D. R. Carneiro M. D. S. Barth A. L. (2021). Y establishing a quantitative index of meropenem hydrolysis for the detection of KPC- and NDM-producing bacteria by MALDI-TOF MS. J. Microbiol. Methods 187:106268. doi: 10.1016/j.mimet.2021.106268, PMID: 34118333

[ref62] Wind L. Keenum I. Gupta S. Ray P. Knowlton K. Ponder M.. (2021). Integrated metagenomic assessment of multiple pre-harvest control points on lettuce resistomes at field-scale. Front. Microbiol. 12:683410. doi: 10.3389/fmicb.2021.683410, PMID: 34305845 PMC8299786

[ref63] Zhong Z. Gao R. Chen Q. Jia L. (2020). Dual-aptamers labeled polydopamine-polyethyleneimine copolymer dots assisted engineering a fluorescence biosensor for sensitive detection of *Pseudomonas aeruginosa* in food samples. Spectrochim. Acta A 224:117417. doi: 10.1016/j.saa.2019.117417, PMID: 31362188

[ref64] Zhou Y. Wan Q. Cai Z. Lu M. Qu X. Wu Q.. (2020). Development and evaluation of loop-mediated isothermal amplification-based kit for rapid detection of *Pseudomonas aeruginosa* in packaged drinking water. Microbiology China 47, 1982–1992. doi: 10.13344/j.microbiol.china.190710

[ref65] Zuccarello L. Barbosa C. Todorovic S. Silveira C. M. (2021). Electrocatalysis by heme enzymes-applications in biosensing. Catalysts 11:218. doi: 10.3390/catal11020218

