# Corrigendum: *Pseudomonas aeruginosa* detection using conventional PCR and quantitative real-time PCR based on species-specific novel gene targets identified by pangenome analysis

**DOI:** 10.3389/fmicb.2025.1583946

**Published:** 2025-04-24

**Authors:** Chufang Wang, Qinghua Ye, Aiming Jiang, Jumei Zhang, Yuting Shang, Fan Li, Baoqing Zhou, Xinran Xiang, Qihui Gu, Rui Pang, Yu Ding, Shi Wu, Moutong Chen, Qingping Wu, Juan Wang

**Affiliations:** ^1^College of Food Science, South China Agricultural University, Guangzhou, China; ^2^Guangdong Provincial Key Laboratory of Microbial Safety and Health, State Key Laboratory of Applied Microbiology Southern China, Institute of Microbiology, Guangdong Academy of Sciences, Guangzhou, China

**Keywords:** novel target gene, *Pseudomonas aeruginosa*, pangenome analysis, PCR, ready-to-eat vegetables

In the published article, there were errors in Figures 2, 3, page 8 as published.

The purpose of both Figures 2, 3 was to explore the sensitivity of the novel identified target detection between genomic DNA and pure culture of *P. aeruginosa*. Unfortunately, during the final uploading of the data, the Figures 2, 3 were pasted incorrectly.

The corrected Figures 2, 3 and their captions appear below.

**Figure 2:**
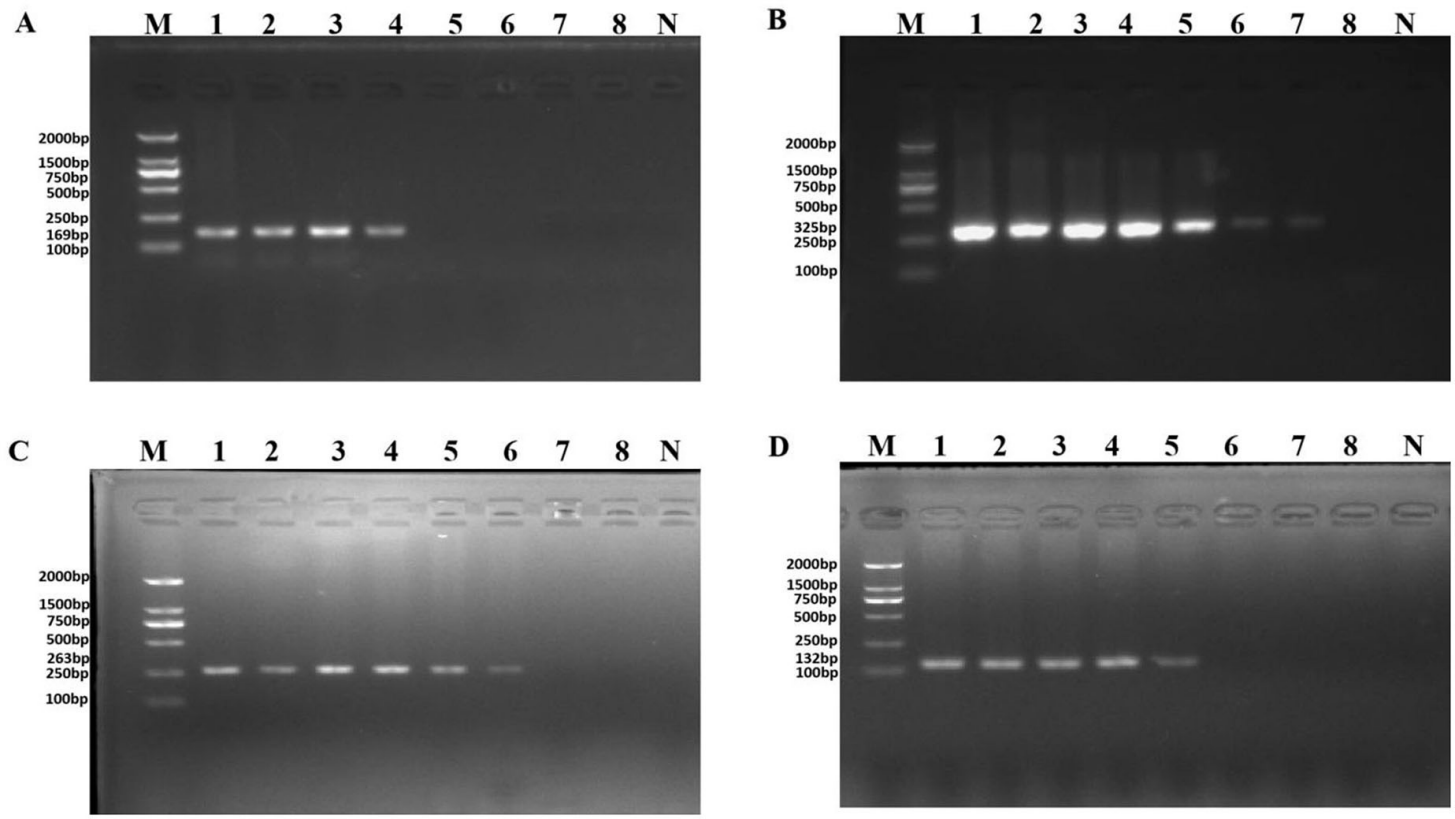
PCR detection sensitivity using dilutions of genomic DNA from *Pseudomonas aeruginosa* ATCC 15442. Lane M = DSTM 2000 marker (Dongsheng Biotechnology, Guangdong, China); lane N = negative control (double-distilled H_2_O); lanes 1–8 = 65.4 ng/μl, 6.54 ng/μl, 654 pg/μl, 65.4 pg/μl, 6.54 pg/μl, 654 fg/μl, and 65.4 fg/μl, 6.54 fg/μl, respectively. **(A)** Primer set PA1 (169 bp); **(B)** primer set PA2 (325 bp); **(C)** primer set PA3 (263 bp); and **(D)** primer set PA4 (132 bp).

**Figure 3:**
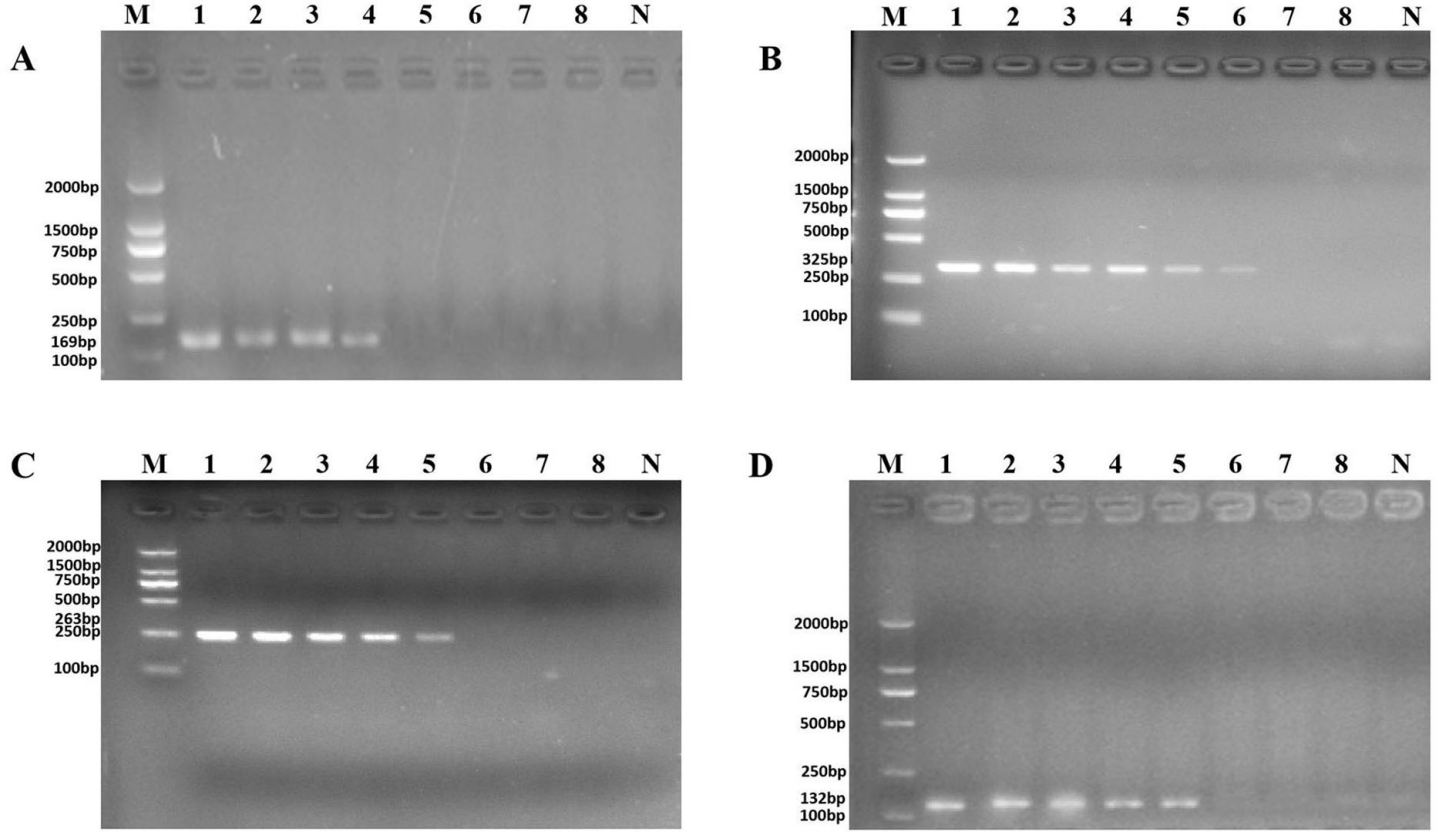
PCR detection sensitivity using dilutions of a pure culture of *P. aeruginosa* ATCC 15442. Lane M = DSTM 2000 marker (Dongsheng Biotechnology, Guangdong, China); lane N = negative control (double-distilled H_2_O); and lanes 1–8 = 2.07 × 10^8^ CFU/ml, 2.07 × 10^7^ CFU/ml, 1.85 × 10^6^ CFU/ml, 4.15 × 10^5^ CFU/ml, 4.3 × 10^4^ CFU/ml, 9.7 × 10^3^ CFU/ml, 1.4 × 10^2^ CFU/ml, and 2 × 10^1^ CFU/ml, respectively. **(A)** Primer set PA1 (169 bp); **(B)** primer set PA2 (325 bp); **(C)** primer set PA3 (263 bp); and **(D)** primer set PA4 (132 bp).

In the published article, there was an error in Supplementary Figure 1. During the assembling of different Figures, we mistakenly pasted Figure S1.d at the position of Figure S1.h.

The authors apologize for this error and state that this does not change the scientific conclusions of the article in any way. The original article has been updated.

