# Supplementary material for: Pseudomonas aeruginosa Detection Using Conventional PCR and Quantitative Real-Time PCR Based on Species-Specific Novel Gene Targets Identified by Pangenome Analysis
Source: Front Microbiol. 2022 May 4;13:820431. doi: 10.3389/fmicb.2022.820431 (PMC9119647; doi:10.3389/fmicb.2022.820431)
Supplement: Supplementary file 1 [file Data_Sheet_1.pdf]

# Supplementary Material

## *Pseudomonas aeruginosa* detection using conventional PCR and quantitative real-time PCR (qPCR) based on species-specific novel gene targets identified by pan-genome analysis

Chufang Wang[1,2 #], Qinghua Ye[1 #], Jumei Zhang [2], Qihui Gu [2], Rui Pang [2], Yu Ding [2], Shi Wu [2], Moutong Chen [2], Youxiong Zhang [2],Shuhong Zhang [2], Qingping Wu [1, 2*], Juan Wang [1,2*]

1. College of Food Science, South China Agricultural University, Guangzhou, 510642, China.
2. Guangdong Provincial Key Laboratory of Microbial Safety and Health, State Key Laboratory of Applied Microbiology Southern China, Institute of Microbiology, Guangdong Academy of Sciences, Guangzhou, China.
3. Key Laboratory of Agricultural Microbiomics and Precision Application,Ministry of Agriculture and Rural Affairs.

[*] **Corresponding author:**

Professor Qingping Wu

E-mail: wuqp203@163.com

Tel: +86-20-87688132; fax: +86-20-87688132

Address: Institute of Microbiology, Guangdong Academy of Sciences, Yard 100#, Xianlie Zhong Road, Yuexiu District, 510070 Guangzhou, P.R.China

Associate Professor Juan Wang

E-mail: wangjuan@scau.edu.cn

Address: College of Food Science, South China Agricultural University, Guangzhou 510642, China

[#] These authors contribute to the manuscript equally.

**Table S1.** Information for *Pseudomonas* spp. and non- *Pseudomonas* spp. isolates analyzed during this Study.

| Organism | Species | Assembly |
|---|---|---|
| *Pseudomonas* spp. | *Pseudomonas aeruginosa* | GCA_000014625.1,GCA_000790105.1,GCA_000795985.1,GCA_001036195.1 |
| | | GCA_000017205.1,GCA_000790115.1,GCA_000796005.1,GCA_001036225.1 |
| | | GCA_000148745.1,GCA_000790155.1,GCA_000796045.1,GCA_001036255.1 |
| | | GCA_000152525.1,GCA_000790185.1,GCA_000796065.1,GCA_001036265.1 |
| | | GCA_000152545.1,GCA_000790205.1,GCA_000796085.1,GCA_001036335.1 |
| | | GCA_000168335.1,GCA_000790215.1,GCA_000796095.1,GCA_001036355.1 |
| | | GCA_000215775.5,GCA_000790245.1,GCA_000796125.1,GCA_001036385.1 |
| | | GCA_000215795.5,GCA_000790265.1,GCA_000796145.1,GCA_001036425.1 |
| | | GCA_000223925.2,GCA_000790285.1,GCA_000796165.1,GCA_001036465.1 |
| | | GCA_000223945.2,GCA_000790305.1,GCA_000796175.1,GCA_001036485.1 |
| | | GCA_000223965.2,GCA_000790345.1,GCA_000796205.1,GCA_001036495.1 |
| | | GCA_000226155.1,GCA_000790355.1,GCA_000796225.1,GCA_001036505.1 |
| | | GCA_000233495.1,GCA_000790385.1,GCA_000796255.1,GCA_001036545.1 |
| | | GCA_000247435.2,GCA_000790405.1,GCA_000796285.1,GCA_001036565.1 |
| | | GCA_000258285.1,GCA_000790425.1,GCA_000796295.1,GCA_001036575.1 |
| | | GCA_000259025.1,GCA_000790445.1,GCA_000796325.1,GCA_001036585.1 |
| | | GCA_000271365.1,GCA_000790465.1,GCA_000796345.1,GCA_001036605.1 |
| | | GCA_000271985.2,GCA_000790485.1,GCA_000796365.1,GCA_001036645.1 |
| | | GCA_000282915.1,GCA_000790505.1,GCA_000796385.1,GCA_001036655.1 |
| | | GCA_000284555.1,GCA_000790525.1,GCA_000796405.1,GCA_001036675.1 |
| | | GCA_000287875.1,GCA_000790545.1,GCA_000796425.1,GCA_001036685.1 |
| | | GCA_000291745.1,GCA_000790605.1,GCA_000796465.1,GCA_001036735.1 |
| | | GCA_000296325.1,GCA_000790625.1,GCA_000796475.1,GCA_001036765.1 |
| | | GCA_000297335.1,GCA_000790645.1,GCA_000796505.1,GCA_001036775.1 |
| | | GCA_000297355.1,GCA_000790655.1,GCA_000796545.1,GCA_001036805.1 |
| | | GCA_000341565.1,GCA_000790685.1,GCA_000796555.1,GCA_001036815.1 |
| | | GCA_000342145.1,GCA_000790705.1,GCA_000796585.1,GCA_001036835.1 |
| | | GCA_000359505.1,GCA_000790725.1,GCA_000796605.1,GCA_001036885.1 |
| | | GCA_000359565.1,GCA_000790735.1,GCA_000796615.1,GCA_001036895.1 |
| | | GCA_000399805.1,GCA_000790765.1,GCA_000796645.1,GCA_001036905.1 |
| | | GCA_000404265.1,GCA_000790785.1,GCA_000796655.1,GCA_001036965.1 |
| | | GCA_000407905.1,GCA_000790805.1,GCA_000796685.1,GCA_001036975.1 |
| | | GCA_000408865.1,GCA_000790825.1,GCA_000796705.1,GCA_001037025.1 |
| | | GCA_000412735.1,GCA_000790835.1,GCA_000796725.1,GCA_001037045.1 |
| | | GCA_000414035.1,GCA_000790865.1,GCA_000796745.1,GCA_001037055.1 |
| | | GCA_000439855.1,GCA_000790885.1,GCA_000796765.1,GCA_001037185.1 |
| | | GCA_000439875.1,GCA_000790915.1,GCA_000796785.1,GCA_001037195.1 |
| | | GCA_000467675.1,GCA_000790935.1,GCA_000796795.1,GCA_001037225.1 |
| | | GCA_000468555.1,GCA_000790965.1,GCA_000796825.1,GCA_001037235.1 |
| | | GCA_000468935.1,GCA_000790985.1,GCA_000796865.1,GCA_001037265.1 |
| | | GCA_000473745.3,GCA_000791005.1,GCA_000796875.1,GCA_001037305.1 |
| | | GCA_000478465.2,GCA_000791025.1,GCA_000796905.1,GCA_001037315.1 |
| | | GCA_000480355.1,GCA_000791035.1,GCA_000796925.1,GCA_001037355.1 |
| | | GCA_000480375.1,GCA_000791065.1,GCA_000796945.1,GCA_001037385.1 |

GCA_000480395.1,GCA_000791085.1,GCA_000796985.1,GCA_001037395.1
GCA_000480415.1,GCA_000791105.1,GCA_000797005.1,GCA_001039325.1
GCA_000480435.1,GCA_000791125.1,GCA_000797025.1,GCA_001042925.1
GCA_000480455.1,GCA_000791155.1,GCA_000797045.1,GCA_001045515.1
GCA_000480475.1,GCA_000791185.1,GCA_000797055.1,GCA_001045685.1
GCA_000480495.1,GCA_000791205.1,GCA_000797085.1,GCA_001051345.1
GCA_000480515.1,GCA_000791225.1,GCA_000797105.1,GCA_001061475.1
GCA_000480535.1,GCA_000791235.1,GCA_000797125.1,GCA_001062995.1
GCA_000480555.1,GCA_000791265.1,GCA_000797145.1,GCA_001063005.1
GCA_000480575.1,GCA_000791275.1,GCA_000797165.1,GCA_001064905.1
GCA_000480595.1,GCA_000791305.1,GCA_000797185.1,GCA_001065035.1
GCA_000480615.1,GCA_000791345.1,GCA_000797225.1,GCA_001065395.1
GCA_000480645.1,GCA_000791365.1,GCA_000797245.1,GCA_001076225.1
GCA_000480665.1,GCA_000791385.1,GCA_000797265.1,GCA_001076855.1
GCA_000480685.1,GCA_000791405.1,GCA_000797285.1,GCA_001077475.1
GCA_000480705.1,GCA_000791425.1,GCA_000797305.1,GCA_001086625.1
GCA_000480725.1,GCA_000791485.1,GCA_000797325.1,GCA_001086635.1
GCA_000480745.1,GCA_000791495.1,GCA_000797345.1,GCA_001086645.1
GCA_000480765.1,GCA_000791525.1,GCA_000797355.1,GCA_001086655.1
GCA_000480785.1,GCA_000791545.1,GCA_000797395.1,GCA_001086725.1
GCA_000480805.1,GCA_000791565.1,GCA_000813565.1,GCA_001086735.1
GCA_000480825.1,GCA_000791585.1,GCA_000816985.1,GCA_001086745.1
GCA_000480845.1,GCA_000791605.1,GCA_000817165.1,GCA_001086755.1
GCA_000480865.1,GCA_000791625.1,GCA_000817865.1,GCA_001086805.1
GCA_000480885.1,GCA_000791635.1,GCA_000820805.1,GCA_001180505.1
GCA_000480905.1,GCA_000791665.1,GCA_000823905.1,GCA_001180605.1
GCA_000480925.1,GCA_000791705.1,GCA_000823925.1,GCA_001180625.1
GCA_000480945.1,GCA_000791725.1,GCA_000823945.1,GCA_001180645.1
GCA_000480965.1,GCA_000791735.1,GCA_000823965.1,GCA_001180665.1
GCA_000480985.1,GCA_000791765.1,GCA_000823985.1,GCA_001180825.1
GCA_000481005.1,GCA_000791785.1,GCA_000824005.1,GCA_001180845.1
GCA_000481025.1,GCA_000791805.1,GCA_000824025.1,GCA_001181045.1
GCA_000481045.1,GCA_000791835.1,GCA_000824045.1,GCA_001181065.1
GCA_000481065.1,GCA_000791855.1,GCA_000824065.1,GCA_001181085.1
GCA_000481085.1,GCA_000791885.1,GCA_000824085.1,GCA_001181145.1
GCA_000481105.1,GCA_000791905.1,GCA_000824105.1,GCA_001181185.1
GCA_000481125.1,GCA_000791915.1,GCA_000824125.1,GCA_001181205.1
GCA_000481145.1,GCA_000791945.1,GCA_000824145.1,GCA_001181245.1
GCA_000481165.1,GCA_000791965.1,GCA_000824165.1,GCA_001181265.1
GCA_000481185.1,GCA_000792005.1,GCA_000824185.1,GCA_001181285.1
GCA_000481205.1,GCA_000792025.1,GCA_000824205.1,GCA_001181445.1
GCA_000481225.1,GCA_000792045.1,GCA_000824225.1,GCA_001181465.1
GCA_000481245.1,GCA_000792065.1,GCA_000824245.1,GCA_001181545.1
GCA_000481265.1,GCA_000792085.1,GCA_000824265.1,GCA_001181685.1
GCA_000481285.1,GCA_000792095.1,GCA_000824285.1,GCA_001181785.1
GCA_000481305.1,GCA_000792115.1,GCA_000824305.1,GCA_001181845.1
GCA_000481325.1,GCA_000792145.1,GCA_000824325.1,GCA_001181865.1
GCA_000481345.1,GCA_000792155.1,GCA_000824345.1,GCA_001280745.1
GCA_000481365.1,GCA_000792165.1,GCA_000824365.1,GCA_001280755.1

| | |
|---|---|
| | GCA_000481385.1,GCA_000792225.1,GCA_000824385.1,GCA_001280765.1 |
| | GCA_000481405.1,GCA_000792245.1,GCA_000824405.1,GCA_001293085.1 |
| | GCA_000481425.1,GCA_000792265.1,GCA_000824425.1,GCA_001294675.1 |
| | GCA_000481445.1,GCA_000792285.1,GCA_000824445.1,GCA_001295275.1 |
| | GCA_000481465.1,GCA_000792305.1,GCA_000824465.1,GCA_001295285.1 |
| | GCA_000481485.1,GCA_000792325.1,GCA_000824485.1,GCA_001295295.1 |
| | GCA_000481505.1,GCA_000792345.1,GCA_000824505.1,GCA_001295345.1 |
| | GCA_000481525.1,GCA_000792365.1,GCA_000824525.1,GCA_001373595.1 |
| | GCA_000481545.1,GCA_000792395.1,GCA_000824545.1,GCA_001373615.1 |
| | GCA_000481565.1,GCA_000792425.1,GCA_000824565.1,GCA_001373655.1 |
| | GCA_000481585.1,GCA_000792445.1,GCA_000824585.1,GCA_001373675.1 |
| | GCA_000481605.1,GCA_000792465.1,GCA_000824605.1,GCA_001373695.1 |
| | GCA_000481625.1,GCA_000792475.1,GCA_000824625.1,GCA_001373715.1 |
| | GCA_000481645.1,GCA_000792505.1,GCA_000824645.1,GCA_001373735.1 |
| | GCA_000481665.1,GCA_000792545.1,GCA_000824665.1,GCA_001373755.1 |
| | GCA_000481685.1,GCA_000792565.1,GCA_000824685.1,GCA_001373775.1 |
| | GCA_000481705.1,GCA_000792575.1,GCA_000829255.1,GCA_001373795.1 |
| | GCA_000481725.1,GCA_000792605.1,GCA_000829275.1,GCA_001373815.1 |
| | GCA_000481745.1,GCA_000792625.1,GCA_000829885.1,GCA_001373835.1 |
| | GCA_000481765.1,GCA_000792635.1,GCA_000937465.2,GCA_001373855.1 |
| | GCA_000481785.1,GCA_000792665.1,GCA_000937495.2,GCA_001373875.1 |
| | GCA_000481805.1,GCA_000792675.1,GCA_000950725.1,GCA_001373895.1 |
| | GCA_000481825.1,GCA_000792705.1,GCA_000952805.1,GCA_001373915.1 |
| | GCA_000481845.1,GCA_000792725.1,GCA_000974565.1,GCA_001373935.1 |
| | GCA_000481865.1,GCA_000792765.1,GCA_000981825.1,GCA_001373955.1 |
| | GCA_000481885.1,GCA_000792805.1,GCA_000982125.1,GCA_001373975.1 |
| | GCA_000481905.1,GCA_000792825.1,GCA_000982155.1,GCA_001374015.1 |
| | GCA_000481925.1,GCA_000792845.1,GCA_000988505.1,GCA_001374035.1 |
| | GCA_000481945.1,GCA_000792855.1,GCA_001007215.1,GCA_001374075.1 |
| | GCA_000481965.1,GCA_000792885.1,GCA_001007275.1,GCA_001374095.1 |
| | GCA_000481985.1,GCA_000792895.1,GCA_001007315.1,GCA_001374115.1 |
| | GCA_000482005.1,GCA_000792925.1,GCA_001007425.1,GCA_001374155.1 |
| | GCA_000482025.1,GCA_000792945.1,GCA_001007475.1,GCA_001374175.1 |
| | GCA_000484495.1,GCA_000792965.1,GCA_001010535.1,GCA_001374195.1 |
| | GCA_000484545.1,GCA_000792975.1,GCA_001010545.1,GCA_001374215.1 |
| | GCA_000496325.1,GCA_000793005.1,GCA_001013395.1,GCA_001374235.1 |
| | GCA_000496455.2,GCA_000793025.1,GCA_001023625.1,GCA_001374275.1 |
| | GCA_000496605.2,GCA_000793045.1,GCA_001023635.1,GCA_001374295.1 |
| | GCA_000496645.1,GCA_000793055.1,GCA_001023675.1,GCA_001374315.1 |
| | GCA_000504485.1,GCA_000793085.1,GCA_001023685.1,GCA_001374355.1 |
| | GCA_000505825.1,GCA_000793105.1,GCA_001023705.1,GCA_001374375.1 |
| | GCA_000506805.1,GCA_000793125.1,GCA_001023755.1,GCA_001374395.1 |
| | GCA_000506885.1,GCA_000793135.1,GCA_001023765.1,GCA_001374415.1 |
| | GCA_000508765.1,GCA_000793165.1,GCA_001023815.1,GCA_001374455.1 |
| | GCA_000510305.1,GCA_000793185.1,GCA_001023835.1,GCA_001374475.1 |
| | GCA_000513235.1,GCA_000793205.1,GCA_001023845.1,GCA_001374495.1 |
| | GCA_000520175.1,GCA_000793225.1,GCA_001023875.1,GCA_001374515.1 |
| | GCA_000520195.1,GCA_000793245.1,GCA_001023895.1,GCA_001374555.1 |
| | GCA_000520215.1,GCA_000793255.1,GCA_001023915.1,GCA_001374575.1 |

GCA_000520235.1,GCA_000793285.1,GCA_001023925.1,GCA_001374595.1
GCA_000520255.1,GCA_000793295.1,GCA_001023975.1,GCA_001374615.1
GCA_000520275.1,GCA_000793315.1,GCA_001023985.1,GCA_001374635.1
GCA_000520295.1,GCA_000793345.1,GCA_001023995.1,GCA_001374655.1
GCA_000520315.1,GCA_000793365.1,GCA_001024035.1,GCA_001374675.1
GCA_000520335.1,GCA_000793385.1,GCA_001024055.1,GCA_001374695.1
GCA_000520355.1,GCA_000793395.1,GCA_001024065.1,GCA_001374715.1
GCA_000520375.1,GCA_000793415.1,GCA_001024075.1,GCA_001374735.1
GCA_000520395.1,GCA_000793445.1,GCA_001024115.1,GCA_001374755.1
GCA_000520415.1,GCA_000793455.1,GCA_001024125.1,GCA_001374775.1
GCA_000520435.1,GCA_000793485.1,GCA_001024135.1,GCA_001374795.1
GCA_000520455.1,GCA_000793505.1,GCA_001024205.1,GCA_001374815.1
GCA_000524595.1,GCA_000793585.1,GCA_001024215.1,GCA_001374835.1
GCA_000531435.1,GCA_000793595.1,GCA_001024275.1,GCA_001374855.1
GCA_000558345.1,GCA_000793615.1,GCA_001024285.1,GCA_001374915.1
GCA_000568115.1,GCA_000793645.1,GCA_001024305.1,GCA_001374935.1
GCA_000568215.1,GCA_000793665.1,GCA_001024365.1,GCA_001374955.1
GCA_000568855.2,GCA_000793685.1,GCA_001024375.1,GCA_001374975.1
GCA_000572265.1,GCA_000793705.1,GCA_001024435.1,GCA_001374995.1
GCA_000583895.1,GCA_000793725.1,GCA_001024445.1,GCA_001375015.1
GCA_000583915.1,GCA_000793745.1,GCA_001024455.1,GCA_001375035.1
GCA_000583935.1,GCA_000793765.1,GCA_001024495.1,GCA_001375055.1
GCA_000583955.1,GCA_000793785.1,GCA_001024515.1,GCA_001375075.1
GCA_000583975.1,GCA_000793805.1,GCA_001024535.1,GCA_001375095.1
GCA_000583995.1,GCA_000793825.1,GCA_001024545.1,GCA_001375115.1
GCA_000611995.2,GCA_000793845.1,GCA_001024565.1,GCA_001375135.1
GCA_000626655.2,GCA_000793865.1,GCA_001024615.1,GCA_001375195.1
GCA_000629025.1,GCA_000793885.1,GCA_001024645.1,GCA_001375215.1
GCA_000629045.1,GCA_000793905.1,GCA_001024655.1,GCA_001375235.1
GCA_000629065.1,GCA_000793915.1,GCA_001024695.1,GCA_001375255.1
GCA_000629085.1,GCA_000793945.1,GCA_001024715.1,GCA_001375275.1
GCA_000629105.1,GCA_000793955.1,GCA_001024725.1,GCA_001375295.1
GCA_000629125.1,GCA_000793985.1,GCA_001024755.1,GCA_001375315.1
GCA_000629145.1,GCA_000794005.1,GCA_001024765.1,GCA_001375335.1
GCA_000629165.1,GCA_000794025.1,GCA_001024775.1,GCA_001375355.1
GCA_000629185.1,GCA_000794045.1,GCA_001024805.1,GCA_001375375.1
GCA_000629205.1,GCA_000794055.1,GCA_001024835.1,GCA_001375395.1
GCA_000629225.1,GCA_000794085.1,GCA_001024845.1,GCA_001375415.1
GCA_000629245.1,GCA_000794165.1,GCA_001024855.1,GCA_001375435.1
GCA_000629265.1,GCA_000794175.1,GCA_001024875.1,GCA_001375455.1
GCA_000629285.1,GCA_000794185.1,GCA_001024925.1,GCA_001402955.1
GCA_000629305.1,GCA_000794195.1,GCA_001024945.1,GCA_001414085.1
GCA_000629325.1,GCA_000794245.1,GCA_001024955.1,GCA_001414105.1
GCA_000629345.1,GCA_000794265.1,GCA_001024995.1,GCA_001414155.1
GCA_000629365.1,GCA_000794285.1,GCA_001028745.1,GCA_001414165.1
GCA_000629385.1,GCA_000794305.1,GCA_001034665.1,GCA_001414175.1
GCA_000629405.1,GCA_000794325.1,GCA_001034675.1,GCA_001420205.1
GCA_000629425.1,GCA_000794335.1,GCA_001034685.1,GCA_001420225.1
GCA_000629445.1,GCA_000794365.1,GCA_001034745.1,GCA_001420525.1

GCA_000629465.1,GCA_000794405.1,GCA_001034755.1,GCA_001420535.1
GCA_000629485.1,GCA_000794425.1,GCA_001034765.1,GCA_001444755.1
GCA_000629505.1,GCA_000794445.1,GCA_001034785.1,GCA_001444765.1
GCA_000629525.1,GCA_000794455.1,GCA_001034825.1,GCA_001444815.1
GCA_000629545.1,GCA_000794485.1,GCA_001034835.1,GCA_001444865.1
GCA_000629565.1,GCA_000794505.1,GCA_001034855.1,GCA_001444895.1
GCA_000629585.1,GCA_000794515.1,GCA_001034875.1,GCA_001444915.1
GCA_000629605.1,GCA_000794555.1,GCA_001034905.1,GCA_001444925.1
GCA_000632755.1,GCA_000794585.1,GCA_001034915.1,GCA_001447845.1
GCA_000633495.1,GCA_000794605.1,GCA_001034945.1,GCA_001449145.1
GCA_000647595.2,GCA_000794665.1,GCA_001034985.1,GCA_001449155.1
GCA_000647615.1,GCA_000794735.1,GCA_001035005.1,GCA_001449165.1
GCA_000647935.3,GCA_000794785.1,GCA_001035025.1,GCA_001449175.1
GCA_000685845.1,GCA_000794825.1,GCA_001035035.1,GCA_001449225.1
GCA_000689435.1,GCA_000794845.1,GCA_001035065.1,GCA_001449245.1
GCA_000698765.1,GCA_000794865.1,GCA_001035075.1,GCA_001449255.1
GCA_000705155.1,GCA_000794945.1,GCA_001035105.1,GCA_001449275.1
GCA_000705175.1,GCA_000794965.1,GCA_001035115.1,GCA_001449305.1
GCA_000705215.1,GCA_000794985.1,GCA_001035145.1,GCA_001449325.1
GCA_000709285.1,GCA_000794995.1,GCA_001035155.1,GCA_001449345.1
GCA_000710625.1,GCA_000795025.1,GCA_001035175.1,GCA_001449365.1
GCA_000714515.1,GCA_000795045.1,GCA_001035225.1,GCA_001449385.1
GCA_000737795.1,GCA_000795065.1,GCA_001035235.1,GCA_001449395.1
GCA_000743405.1,GCA_000795085.1,GCA_001035245.1,GCA_001449425.1
GCA_000751715.1,GCA_000795105.1,GCA_001035305.1,GCA_001449435.1
GCA_000757505.1,GCA_000795115.1,GCA_001035325.1,GCA_001449465.1
GCA_000760495.2,GCA_000795145.1,GCA_001035395.1,GCA_001449485.1
GCA_000760505.2,GCA_000795165.1,GCA_001035415.1,GCA_001449505.1
GCA_000763245.3,GCA_000795205.1,GCA_001035445.1,GCA_001449515.1
GCA_000786485.1,GCA_000795225.1,GCA_001035465.1,GCA_001449555.1
GCA_000786565.1,GCA_000795235.1,GCA_001035495.1,GCA_001449585.1
GCA_000789485.1,GCA_000795285.1,GCA_001035535.1,GCA_001449605.1
GCA_000789495.1,GCA_000795305.1,GCA_001035565.1,GCA_001449625.1
GCA_000789525.1,GCA_000795325.1,GCA_001035575.1,GCA_001449675.1
GCA_000789535.1,GCA_000795345.1,GCA_001035605.1,GCA_001449705.1
GCA_000789545.1,GCA_000795365.1,GCA_001035635.1,GCA_001449725.1
GCA_000789605.1,GCA_000795385.1,GCA_001035645.1,GCA_001449735.1
GCA_000789625.1,GCA_000795405.1,GCA_001035685.1,GCA_001449765.1
GCA_000789635.1,GCA_000795425.1,GCA_001035705.1,GCA_001449785.1
GCA_000789645.1,GCA_000795465.1,GCA_001035715.1,GCA_001449805.1
GCA_000789685.1,GCA_000795485.1,GCA_001035725.1,GCA_001449825.1
GCA_000789705.1,GCA_000795525.1,GCA_001035765.1,GCA_001449835.1
GCA_000789725.1,GCA_000795535.1,GCA_001035785.1,GCA_001449865.1
GCA_000789745.1,GCA_000795565.1,GCA_001035795.1,GCA_001449885.1
GCA_000789755.1,GCA_000795585.1,GCA_001035805.1,GCA_001449905.1
GCA_000789785.1,GCA_000795605.1,GCA_001035865.1,GCA_001449925.1
GCA_000789815.1,GCA_000795625.1,GCA_001035875.1,GCA_001449935.1
GCA_000789835.1,GCA_000795635.1,GCA_001035885.1,GCA_001449965.1
GCA_000789885.1,GCA_000795685.1,GCA_001035925.1,GCA_001449985.1

| | |
|---|---|
| | GCA_000789905.1,GCA_000795705.1,GCA_001035945.1,GCA_001450005.1 |
| | GCA_000789925.1,GCA_000795725.1,GCA_001035955.1,GCA_001450015.1 |
| | GCA_000789935.1,GCA_000795735.1,GCA_001035975.1,GCA_001450045.1 |
| | GCA_000789965.1,GCA_000795765.1,GCA_001036005.1,GCA_001450065.1 |
| | GCA_000789975.1,GCA_000795785.1,GCA_001036025.1,GCA_001450085.1 |
| | GCA_000789995.1,GCA_000795815.1,GCA_001036105.1,GCA_001450095.1 |
| | GCA_000790025.1,GCA_000795845.1,GCA_001036115.1,GCA_001450115.1 |
| | GCA_000790035.1,GCA_000795875.1,GCA_001036145.1,GCA_001450165.1 |
| | GCA_000790065.1,GCA_000795925.1,GCA_001036155.1,GCA_001450185.1 |
| | GCA_000790085.1,GCA_000795945.1,GCA_001036185.1,GCA_001450195.1 |
| *Pseudomonas syringae* | GCA_000012245.1,GCA_000344335.2,GCA_000344355.2,GCA_000344475.3, |
| | GCA_000452445.3,GCA_000452705.3,GCA_000648735.3,GCA_000988395.1, |
| | GCA_000988485.1,GCA_001281365.1,GCA_001482725.1,GCA_001913215.1, |
| | GCA_001913235.1,GCA_002024285.1,GCA_002024305.1,GCA_002763655.1, |
| | GCA_002905815.2,GCA_003047185.1,GCA_003665415.1,GCA_004006335.1, |
| | GCA_900235815.1,GCA_900235825.1,GCA_900235835.1,GCA_900235865.1, |
| | GCA_900235885.1,GCA_900289125.1, |
| *Pseudomonas putida* | GCA_000007565.2,GCA_000016865.1,GCA_000019125.1,GCA_000019445.1, |
| | GCA_000183645.1,GCA_000219705.1,GCA_000226035.3,GCA_000264665.2, |
| | GCA_000271965.2,GCA_000281215.1,GCA_000325725.1,GCA_000410575.1, |
| | GCA_000412675.1,GCA_000495455.2,GCA_000498395.3,GCA_000691565.1, |
| | GCA_001515585.2,GCA_001636055.1,GCA_001767335.1,GCA_001886975.1, |
| | GCA_002025705.1,GCA_002356095.1,GCA_002736045.1,GCA_002736125.1, |
| | GCA_002741075.1,GCA_003228315.1,GCA_003290365.1,GCA_003671955.1, |
| | GCA_900636645.1, |
| *Pseudomonas fluorescens* | GCA_000237065.1,GCA_000009225.1,GCA_000012445.1,GCA_000262325.2, |
| | GCA_000293885.3,GCA_000730425.1,GCA_000934565.1,GCA_000963495.1, |
| | GCA_001307155.1,GCA_001307275.1,GCA_001708445.1,GCA_001708465.1, |
| | GCA_001708485.1,GCA_001747385.1,GCA_002865505.1,GCA_003055645.1, |
| | GCA_003410335.1,GCA_003612935.1,GCA_003626995.1,GCA_004683905.1, |
| | GCA_900475215.1,GCA_900636635.1,GCA_900636825.1, |
| *Pseudomonas amygdali* | GCA_002068135.1,GCA_002905685.2, |
| *Pseudomonas Pyringae-group-gen* | GCA_000007805.1,GCA_002966555.1,GCA_900235905.1, |
| *Pseudomonas stutzeri* | GCA_000590475.1,GCA_000219605.1,GCA_000013785.1,GCA_000195105.1, |
| | GCA_000267545.1,GCA_000279165.1,GCA_000327065.1,GCA_000661915.1, |
| | GCA_001038645.1,GCA_001648195.1,GCA_003001655.1,GCA_003008495.1, |
| | GCA_003047145.2,GCA_003952645.1,GCA_003952685.1,GCA_005844005.1, |
| | GCA_900636845.1,GCA_900638035.1, |
| *Pseudomonas chlororaphis* | GCA_000698865.1,GCA_000761195.1,GCA_000963835.1,GCA_001023535.1, |
| | GCA_001602135.1,GCA_002355875.1,GCA_002844145.1,GCA_003008635.1, |
| | GCA_003850345.1,GCA_003850365.1,GCA_003850385.1,GCA_003850405.1, |
| | GCA_003850425.1,GCA_003850445.1,GCA_003850465.1,GCA_003850485.1, |
| | GCA_003850505.1,GCA_003850525.1,GCA_003850585.1,GCA_003850605.1, |
| | GCA_003851145.1,GCA_003851165.1,GCA_003851205.1,GCA_003851225.1, |
| | GCA_003851265.1,GCA_003851305.1,GCA_003851345.1,GCA_003851365.1, |
| | GCA_003851385.1,GCA_003851405.1,GCA_003851425.1,GCA_003851445.1, |
| | GCA_003851785.1,GCA_003851805.1,GCA_003851835.1,GCA_003851865.1, |

| | |
|---|---|
| | GCA_003851905.1,GCA_003851925.1,GCA_003851955.1,GCA_003851985.1, GCA_003852005.1,GCA_003945765.1,GCA_900636995.1,GCA_003851285.1, |
| *Pseudomonas savastanoi* | GCA_000012205.1,GCA_000164015.3,GCA_000016565.1,GCA_000204295.1, GCA_000733715.2,GCA_002442555.1,GCA_003008615.1,GCA_900636545.1, |
| *Pseudomonas mendocina* | GCA_000016565.1,GCA_000204295.1,GCA_000733715.2,GCA_002442555.1, GCA_003008615.1,GCA_900636545.1, |
| *Pseudomonas fuscovaginae* | GCA_900108595.1, |
| *Pseudomonas oleovorans* | GCA_000297075.2,GCA_000953455.1, |
| *Pseudomonas avellanae* | GCA_002905795.2, |
| *Pseudomonas mandelii* | GCA_000257545.3, |
| *Pseudomonas monteilii* | GCA_001534745.1,GCA_000510285.1,GCA_000510325.1,GCA_002943995.1, GCA_003671975.1, |
| *Pseudomonas alcaligenes* | GCA_001597285.1, |
| *Pseudomonas fragi* | GCA_001543265.1,GCA_002128325.1, |
| *Pseudomonas brassicacearum* | GCA_000194805.1,GCA_000585995.1,GCA_001449085.1,GCA_001746815.1, |
| *Pseudomonas veronii* | GCA_002028325.1,GCA_004919535.1,GCA_900092355.1, |
| *Pseudomonas-resinovorans* | GCA_000412695.1, |
| *Pseudomonas protegens* | GCA_000397205.1,GCA_000012265.1,GCA_000828695.1,GCA_002006545.1, GCA_002208745.2,GCA_003363755.1,GCA_900560965.1, |
| *Pseudomonas-alcaliphila* | GCA_001941865.1, |
| *Pseudomonas synxantha* | GCA_000968415.2,GCA_003851465.1,GCA_003851495.1,GCA_003851525.1, GCA_003851555.1,GCA_003852025.1, |
| *Pseudomonas fulva* | GCA_000213805.1,GCA_002688705.1,GCA_002951475.1, |
| *Pseudomonas entomophila* | GCA_000026105.1,GCA_003940785.1,GCA_003940825.1, |
| *Pseudomonas balearica* | GCA_000818015.1, |
| *Pseudomonas corrugata* | GCA_001708425.1, |
| *Pseudomonas cichorii* | GCA_000517305.1, |
| *Pseudomonas mosselii* | GCA_002309555.1,GCA_002736065.1, |
| *Pseudomonas poae* | GCA_000336465.1,GCA_004000515.1, |
| *Pseudomonas parafulva* | GCA_000800255.1,GCA_002021815.1,GCA_003410295.1, |
| *Pseudomonas oryzihabitans* | GCA_001518815.1, |
| *Pseudomonas mucidolens* | GCA_900475945.1, |
| *Pseudomonas cremoricolorata* | GCA_000759535.1, |
| *Pseudomonas citronellolis* | GCA_001654435.1,GCA_001586155.1, |
| *Pseudomonas azotoformans* | GCA_001579805.1,GCA_002007785.1, |
| *Pseudomonas psychrotolerans* | GCA_001913135.1, |
| *Pseudomonas Soli* | GCA_000498975.2, |
| *Pseudomonas arsenicoxydans* | GCA_004135995.1,GCA_900103875.1, |
| *Pseudomonas antarctica* | GCA_001647715.1, |
| *Pseudomonas koreensis* | GCA_001605965.1,GCA_001654515.1,GCA_003049825.1, |
| *Pseudomonas orientalis* | GCA_002934065.1,GCA_003851585.1,GCA_003851605.1,GCA_003851645.1, GCA_003852045.1, |
| *Pseudomonas libanensis* | GCA_003952245.1, |
| *Pseudomonas trivialis* | GCA_001186335.1, |
| *Pseudomonas taetrolens* | GCA_900475,GCA_900637 |
| *Pseudomonas frederiksbergensis* | GCA_001874645.1,GCA_001952935.1,GCA_002355315.1, |
| *Pseudomonas lundensis* | GCA_001020725.2, |
| *Pseudomonas kribbensis* | GCA_003352185.1, |

| | | |
|---|---|---|
| | *Pseudomonas furukawaii* | GCA_002355475.1, |
| | *Pseudomonas lurida* | GCA_002966835.1, |
| | *Pseudomonas verPuta* | GCA_001294575.1, |
| | *Pseudomonas yamanorum* | GCA_001612705.2, |
| | *Pseudomonas ceraPi* | GCA_900074915.1,GCA_900235895.1, |
| | *Pseudomonas alkylphenolica* | GCA_000746525.1, |
| | *Pseudomonassp.ADP* | GCA_001465445.1, |
| Non-*Pseudomonas.spp.* | *Brevundimonas diminuta* | GCA_004102925.1, |
| | *Micavibrio aeruginosavorus* | *GCA_000226315.1,GCA_000348745.1,* |
| | *YerPinia enterocolitica* | GCA_000987925.1, |
| | *Bacillus cereus* | GCA_006094295.1, |
| | *Staphylococcus aureus* | GCA_000011265.1,GCA_000189455.3,GCA_000237125.1,GCA_000487775.2, GCA_000017125.1,GCA_001611385.1,GCA_001656075.1,GCA_000748105.1, GCA_900474555.1,GCA_900092595.1,GCA_001580495.1,GCA_000749295.1, GCA_003354545.1,GCA_002850395.1,GCA_000013425.1,GCA_001245445.1, GCA_900636335.1,GCA_000160335.2,GCA_000239235.1,GCA_001247475.1, GCA_001611425.1,GCA_000011505.1,GCA_003030225.1,GCA_001305835.1, GCA_002803885.1,GCA_000009585.1,GCA_900474665.1,GCA_001317685.2, GCA_002140115.1,GCA_000237265.1,GCA_000005845.2,GCA_001481155.1, GCA_000284535.1,GCA_003030085.1,GCA_000006945.2,GCA_001534905.1, GCA_000025145.2,GCA_003030065.1,GCA_000007545.1,GCA_003117335.1, GCA_000383005.1,GCA_001594205.1,GCA_000008105.1,GCA_900185485.1, GCA_000204665.1,GCA_000159535.2,GCA_000171535.2,GCA_000487615.2, GCA_900474575.1,GCA_001548295.1,GCA_000487515.2,GCA_000462955.1, GCA_003264775.1,GCA_000009645.1,GCA_000487575.2,GCA_000756205.1. |
| | *Escherichia coli* | GCA_003018455.1 |
| | *Listeria monocytogenes* | GCA_900187225.1 |
| | *salmonella enterica* | GCA_001558355.2 |
| | *Campylobacter jejuni* | GCA_003999645.1 |
| | *Cronobacter sakazakii* | GCA_000982825.1 |
| | *Escherichia coli* | GCA_003697165.2 |
| | *higella sonnei* | GCA_013374815.1 |
| | *Vibrio parahaemolyticus* | GCA_001558495.2 |

**Table S2.** Information for *P.*aeruginosa and non-*P.*aeruginosa for Species-special target analysis in this study.

| No. | Bacterial species | strains numbner | Number of PtrainP | Pource* | species-special target for PCR or qPCR assay | |
|---|---|---|---|---|---|---|
| | | | | | ***PCR*** | **qPCR** |
| 1 | *P. aeruginosa* | ATCC27853 | 1 | a | + | + |
| 2 | *P. aeruginosa* | ATCC9027 | 1 | a | + | + |
| 3 | *P. aeruginosa* | ATCC15442 | 1 | a | + | + |
| 4 | *P. aeruginosa* | GIM1.46 | 1 | b | + | + |
| 5 | *P. aeruginosa* | Laboratory isolate | 91 | a | + | (59)+ |
| 6 | *P.putida* | *ST25-10* | 1 | a | - | - |
| 7 | *P.putida* | *GIM1.57* | 1 | b | - | - |
| 8 | *P.fuscovaginae* | *ST42-2* | 1 | a | - | - |
| 9 | *P.hunanensis* | *0617-8* | 1 | a | - | - |
| 10 | *P.fulva* | *0625-4* | 1 | a | - | - |
| 11 | *P.kilonensis* | *ST38-5* | 1 | a | - | - |
| 12 | *P.lini* | *M41023-1* | 1 | a | - | - |
| *13* | *P.jessenii* | *ST42-4* | 1 | a | - | - |
| 14 | *P.Alcaligenes* | *CMCC1.1806* | 1 | b | - | - |
| 15 | *P.chlororaphis* | *1143-3* | 1 | a | - | - |
| 16 | *P.fragi* | *52532-7* | 1 | a | - | - |
| 17 | *P.Mendoza* | *CMCC1.1804* | 1 | b | - | - |
| 18 | *P.mosselii* | *ST42-10* | 1 | a | - | - |
| 19 | *P.corrugata* | *ST19-4* | 1 | a | - | - |
| 20 | *P.oleovorans* | *M43075-4* | 1 | a | - | - |
| 21 | *P.taiwanensis* | *0617-3* | 1 | a | - | - |
| 22 | *P.geniculata* | *52023-3* | 1 | a | - | - |
| *23* | *P.fluorescens* | *51184-3* | 1 | a | - | - |
| *24* | *P.fluorescens* | *GIM1.492* | 1 | b | - | - |
| *25* | *E.coli* | *ATCC 25922* | 1 | a | - | - |
| *26* | *E.coli* | *1656-1* | 1 | a | - | - |
| *27* | *S.hominis* | *1006-1* | 1 | a | - | - |
| *28* | *S.hominis* | *0656-4* | 1 | a | - | - |
| *29* | *S.haemolyticus* | *0620* | 1 | a | - | - |
| *30* | *Y. enterocolitica* | *Y1408* | 1 | a | - | - |
| *31* | *Y. enterocolitica* | *C009* | 1 | a | - | - |
| *32* | *Y. enterocolitica* | *Y2602* | 1 | a | - | - |

| | | | | | | |
|---|---|---|---|---|---|---|
| *33* | *Y. enterocolitica* | *Y3553* | 1 | a | - | - |
| *34* | *L. monocytogenes* | *1333-2* | 1 | a | - | - |
| *35* | *L. monocytogenes* | *2545-2* | 1 | a | - | - |
| *36* | *L. monocytogenes* | *509A1-3* | 1 | a | - | - |
| *37* | *E.coli* | *1679* | 1 | a | - | - |
| *38* | *E.coli* | *1677-3* | 1 | a | - | - |
| *39* | *S.epidermis* | *0597* | 1 | a | - | - |
| *40* | *B.cereus* | *1378* | 1 | a | - | - |
| *41* | *B.cereus* | *wqr5* | 1 | a | - | - |
| *42* | *S.aureus* | *800* | 1 | a | - | - |
| *43* | *Salmonella* | *839* | 1 | a | - | - |
| *44* | *Salmonella* | *838* | 1 | a | - | - |

[*] a, our laboratory; b, Guangdong Huankai Co., Ltd., China

[1] ATCC, American Type Culture Collection, USA.

[2] CMCC, China Medical Culture Collection, China.

[3] /, Laboratory isolate. Result (+/-) indicate positive and negative signals.

**Table S3.** Species-specific target and primers for PCR and qPCR identification of pathogenic *P.aeruginosa*.

| Species | Name of target genes | Encoded protein | Primer set name | Sequences (5'-3') | Product Size (bp) | For PCR or qPCR assay |
|---|---|---|---|---|---|---|
| *P. aeruginosa* | *group_98983* | hypothetical protein | PA1 | CTCCGTGGAAAAGCAGTTG<br>GCGTATGCCGACGTAGAAT | 169 | PCR |
| | *phzA2* | Phenazine biosynthesis protein PhzA2 | PA2 | GTTTACCGACAACCTGGAA<br>GCAATAGCCCTGCGGATAC | 325 | PCR |
| | | | PA12 | CAACTGGACCACGGAAAGC<br>GTCTCGAAGATCCGCACGT | 126 | qPCR |
| | *group_75393* | hypothetical protein | PA3 | ATGGACAGGGACGCATTGA<br>CGAGGGACGAAGGTAAGGA | 263 | PCR |
| | *group_88276* | hypothetical protein | PA4 | GACTCTACCCTCCCTGACTT<br>TCCATCACCGAGAAGC | 132 | PCR<br>PCR |
| | *group_91687* | hypothetical protein | PA5 | TTGGCAAGGTGCGGCGGGAGT<br>AGGCAGCCGTTCCACCCGTCC | *345* | PCR<br>PCR |
| | *group_103885* | Putative S-adenosyl-L-methionine-dependent methyltransferase | PA6 | TGATGCCTTATCTGCGGGAGT<br>GGAAGGCGAAGCCGTTGTA | *306* | PCR<br>PCR |
| | *hcpC_1* | Putative beta-lactamase HcpC | PA7 | ATGCGGCTGATGGCTATTG<br>CGCTCCACTGCCTTACTGT | 221 | PCR<br>PCR |
| | *group_95405* | hypothetical protein | PA8 | TACGGCTTTCCCAGGCAGG<br>GGGCGGAGTTGAGTTGTTG | 237 | PCR<br>PCR |
| | *epsM* | Type II secretion system protein M | PA9 | CTGCTCTGGCTCGGTGCGT<br>CCCTGCCCGTTCAACTGCT | *344* | PCR<br>PCR |
| | *group_66058* | hypothetical protein | PA10 | ACCTCTGTCGCTACCATCA<br>CGGATGAACTCGACCTCTT | *413* | PCR<br>PCR |
| | *sycN* | Chaperone protein SycN | PA11 | ACCTGCGGGTGAGCGT<br>GGCTTCCAGGGTGATGC | 254 | PCR |
| | *phzA2* | Phenazine biosynthesis protein PhzA2 | PA12 | CAACTGGACCACGGAAAGC<br>GTCTCGAAGATCCGCACGT | 126 | qPCR |

**Table S4.** Target of *toxA* gene and primer imformations for PCR identification of *P.aeruginosa*.

| **Name of target genes** | **Gene sequences** |
|---|---|
| forward primer | 5’-ACATCAAGGTGTTCATCC-3’ |
| forward primer | 5’-AGGGCACGCTCGTTAGCC-3’ |
| toxA | GACAACGCCCTCAGCATCACCAGCGACGGCCTGACCATCC GCCTCGAAGGCGGCGTCGAGCCGAACAAGCCGGTGCGCT ACAGCTACACGCGCCAGGCGCGCGGCAGTTGGTCGCTGAA CTGGCTGGTGCCGATCGGCCACGAGAAGEETTCGAACATC AAGGTGTTCATCCACGAATGAACGCCGGTAACCAGCTCAG CCACATGTCGCCGATCTACACCATCGAGATGGGCGACGAG TTGCTGGCGAAGCTGGCGCGCGATGCCACCTTCTTCGTCA GGGCGCACGAGAGCAACGAGATGCAGCCGACGCTCGCCA TCAGCCATGCCGGGGTCAGCGTGGTCATGGCCCAGGCCCA GCCGCGCCGGGAAAGCGCTGGAGCGAATGGGCCAGCG |

*Sedighe Rashno Taee et al. ,2014*

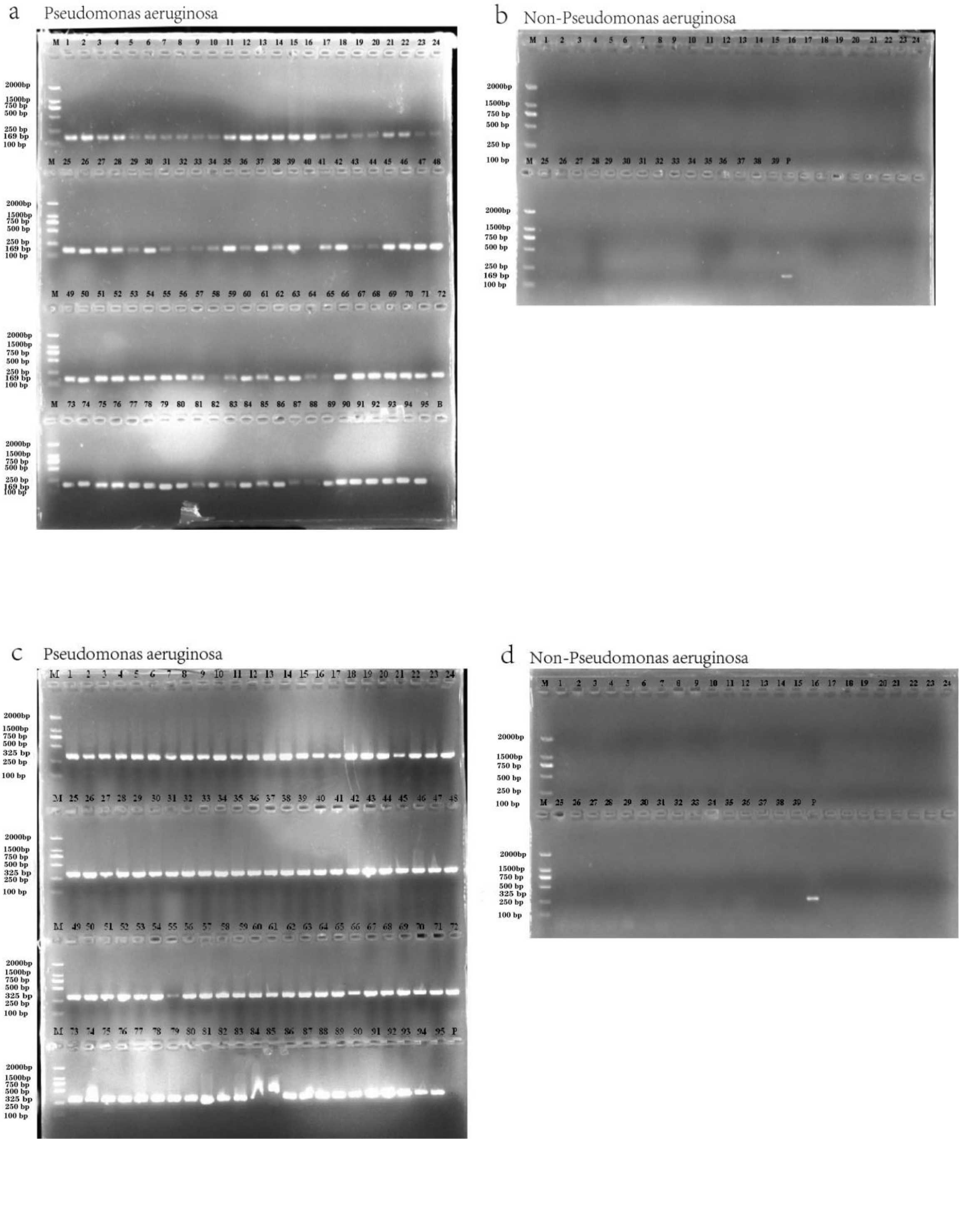
a Pseudomonas aeruginosa
b Non-Pseudomonas aeruginosa
c Pseudomonas aeruginosa
d Non-Pseudomonas aeruginosa
M
2000bp
1500bp
750 bp
500 bp
250 bp
169 bp
100 bp
325 bp
P
B

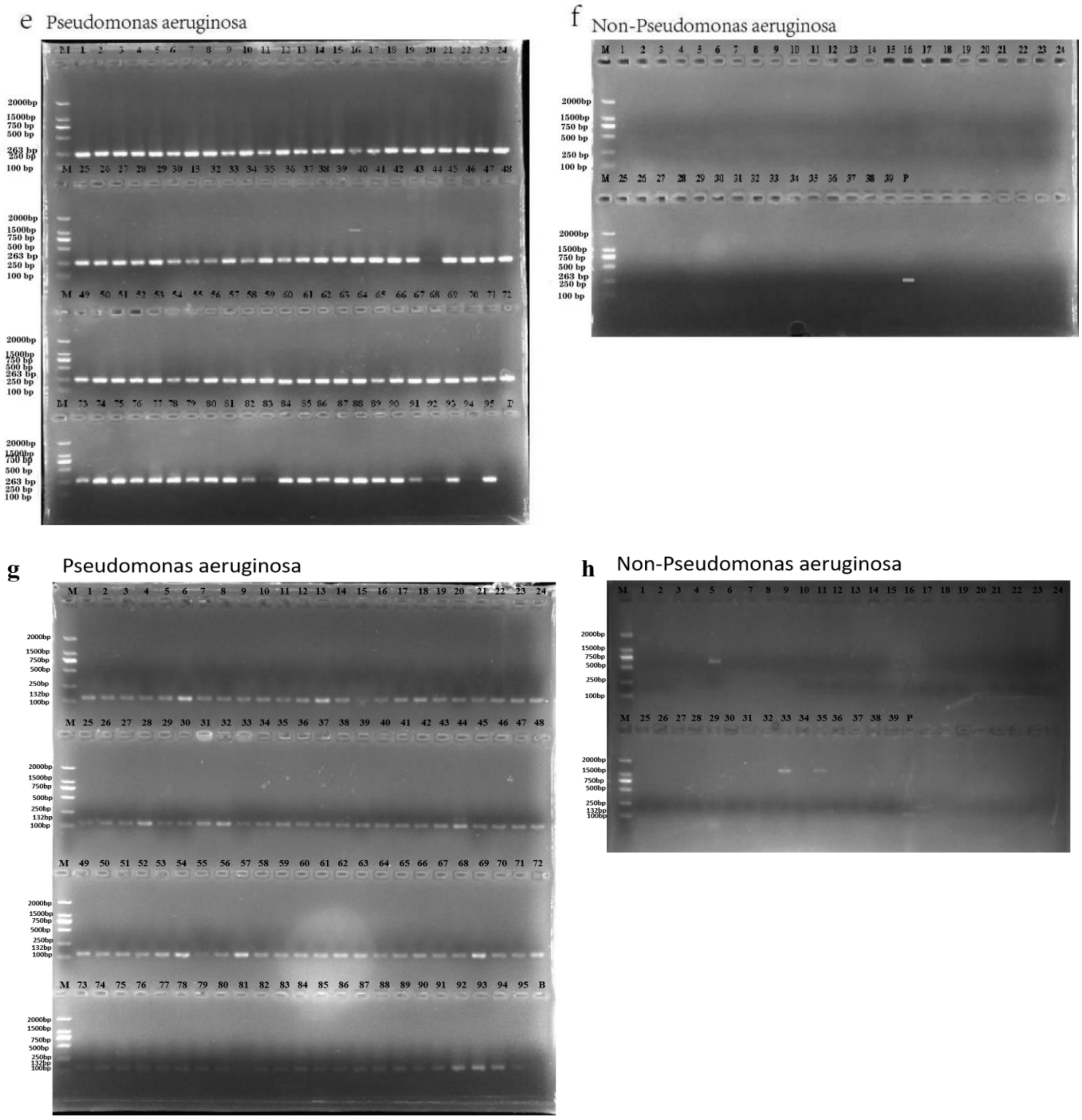


**Figure S1.** Results of PCR of genome DNA of the strains with in the *P.aeruginosa* genus using primer sets PA1 (169bp), PA2 (325bp), PA3 (263bp), and PA4 (132bp). Lane M = DSTM 2000 marker (Dongsheng Biotechnology, Guangdong, China); lane B = negative control (double-distilled H2O);lane P=positive control (*P.aeruginosa* ATCC 9027).

(a) Primer set PA1 *P.aeruginosa*; (b) primer set PA1 non-*P.aeruginosa;*

(c) primer set PA2 *P.aeruginosa* ; (d) primer set PA2 non-*P.aeruginosa* .

(e) primer set PA3 *P.aeruginosa* ; (f) primer set PA3 non-*P.aeruginosa*.

(g) primer set PA4 *P.aeruginosa* ; (h) primer set PA4 non-*P.aeruginosa*.

All concentrations of genomic DNA were diluted to about 20 ng/μL.

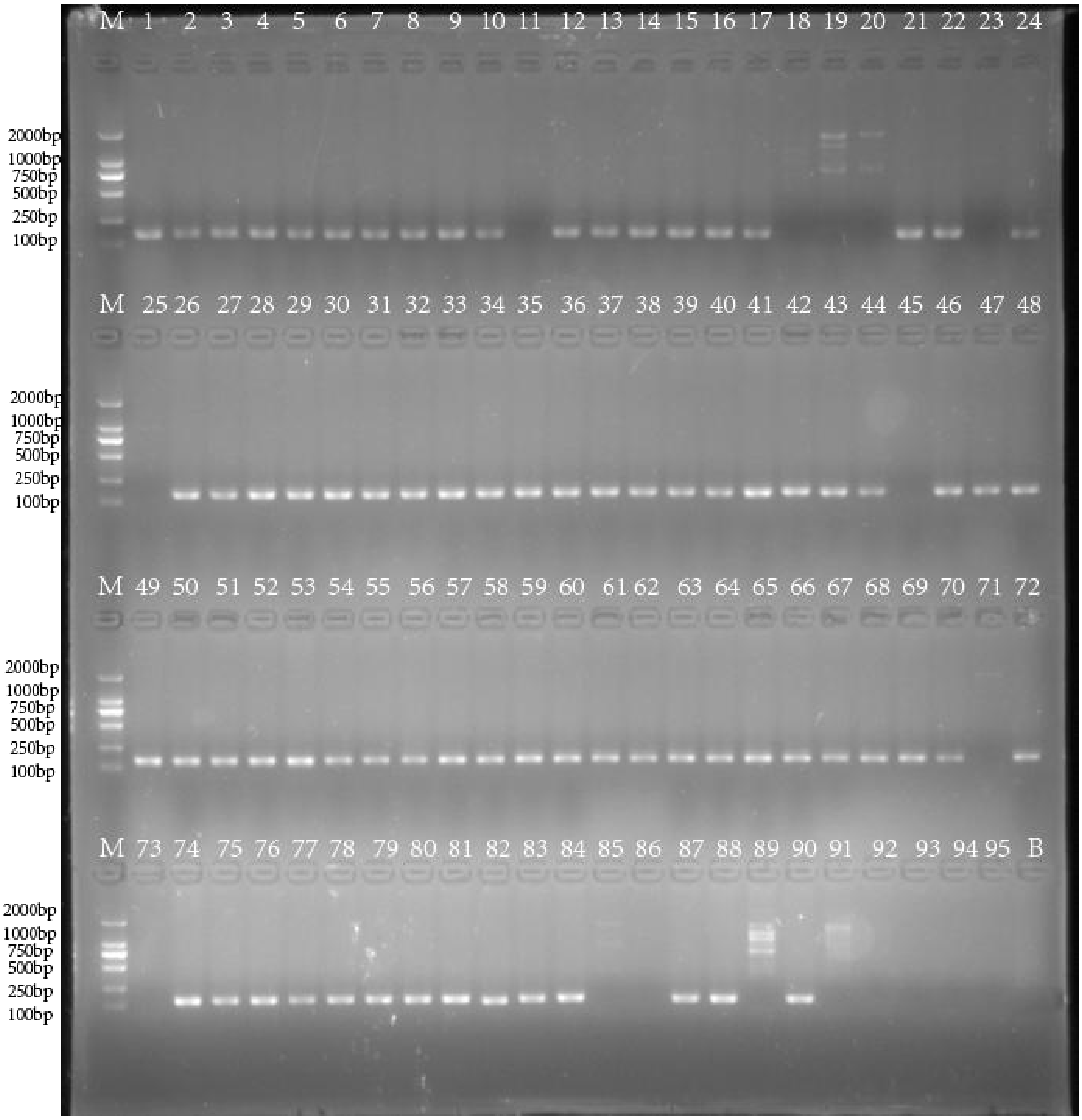


**Figure S2.** Results of PCR of genome DNA of the strains with in the *P.aeruginosa* genus using primer sets toxA gene. Lane M = DSTM 2000 marker (Dongsheng Biotechnology, Guangdong, China); Lane B = negative control (double-distilled $H_2O$). Lane 1-95:*P.aeruginosa* strains.
